# Rapid coupling between gravitational forces and the transcriptome in human myelomonocytic U937 cells

**DOI:** 10.1038/s41598-018-31596-y

**Published:** 2018-09-05

**Authors:** Cora S. Thiel, Svantje Tauber, Swantje Christoffel, Andreas Huge, Beatrice A. Lauber, Jennifer Polzer, Katrin Paulsen, Hartwin Lier, Frank Engelmann, Burkhard Schmitz, Andreas Schütte, Christiane Raig, Liliana E. Layer, Oliver Ullrich

**Affiliations:** 1 Institute of Anatomy, Faculty of Medicine, University of Zurich, Winterthurerstrasse 190, 8057 Zurich, Switzerland; 2 Department of Machine Design, Engineering Design and Product Development, Institute of Mechanical Engineering, Otto-von-Guericke-University Magdeburg, Universitätsplatz 2, 39106 Magdeburg, Germany; 3 Core Facility Genomic, Medical Faculty of Muenster, University of Muenster, Albert-Schweitzer-Campus 1, D3, Domagstrasse 3, 48149 Muenster, Germany; 4 KEK GmbH, Kemberger Str. 5, 06905 Bad Schmiedeberg, Germany; 5 Ernst-Abbe-Hochschule Jena, Carl-Zeiss-Promenade 2, 07745 Jena, Germany; 6 Airbus DS GmbH, Airbus-Allee 1, 28199 Bremen, Germany; 7 Zurich Center for Integrative Human Physiology (ZIHP), University of Zurich, Winterthurerstrasse 190, 8057 Zurich, Switzerland

## Abstract

The gravitational force has been constant throughout Earth’s evolutionary history. Since the cell nucleus is subjected to permanent forces induced by Earth’s gravity, we addressed the question, if gene expression homeostasis is constantly shaped by the gravitational force on Earth. We therefore investigated the transcriptome in force-free conditions of microgravity, determined the time frame of initial gravitational force-transduction to the transcriptome and assessed the role of cation channels. We combined a parabolic flight experiment campaign with a suborbital ballistic rocket experiment employing the human myelomonocytic cell line U937 and analyzed the whole gene transcription by microarray, using rigorous controls for exclusion of effects not related to gravitational force and cross-validation through two fully independent research campaigns. Experiments with the wide range ion channel inhibitor SKF-96365 in combination with whole transcriptome analysis were conducted to study the functional role of ion channels in the transduction of gravitational forces at an integrative level. We detected profound alterations in the transcriptome already after 20 s of microgravity or hypergravity. In microgravity, 99.43% of all initially altered transcripts adapted after 5 min. In hypergravity, 98.93% of all initially altered transcripts adapted after 75 s. Only 2.4% of all microgravity-regulated transcripts were sensitive to the cation channel inhibitor SKF-96365. Inter-platform comparison of differentially regulated transcripts revealed 57 annotated gravity-sensitive transcripts. We assume that gravitational forces are rapidly and constantly transduced into the nucleus as omnipresent condition for nuclear and chromatin structure as well as homeostasis of gene expression.

## Introduction

The gravitational force has been constant throughout the 4 billion years of Earth’s evolutionary history^1^ and played a crucial role in the evolutionary expansion of organisms^2^. All terrestrial life has adapted to this fundamental force by developing structures and functions at the levels of organisms, tissues, cells and molecular systems^3^, probably including the nucleus, chromatin organization and gene expression^4^. While cellular structures are under permanent force transmission in Earth’s gravitational field, the low-force environment of microgravity has been demonstrated to have profound effects at the cellular and molecular level^5^. Gravitational forces may be experienced by an individual cell as a whole^6^ and are transduced as physical force into subcellular structures through the cellular^6–8^ and nuclear architecture^9–11^, altering nuclear plasticity, chromatin organization and accessibility and subsequently gene expression^12–16^. Small forces in the low piconewton range may finally trigger nuclear mechanotransduction^17^ and force transduction into the chromatin can occur within seconds^12^. Thus, the nucleus is subjected to permanent small direct or indirect^18^ forces induced by Earth’s gravity, raising the fundamental question, if gene expression homeostasis is constantly shaped by the gravitational force on Earth.

Only experiments in microgravity allow to investigate gene expression under force-free conditions and therefore facilitate the elucidation of the role of Earth’s gravity in gene expression homeostasis, while time-resolved studies would help to assess the adaptation potential in an altered gravitational environment. Microarray-based gene expression studies have been conducted previously with T cells or T cell lines in simulated microgravity^19–22^, in spaceflight experiments^23–25^, and with lymphatic tissue from space-flown animals^26,27^. As a result, altered expression of microRNA in simulated microgravity conditions^21^ correlated with the gene expression pattern of the transcription factor Rel^21^, which has been identified as microgravity-dependent gene expression regulator in a spaceflight experiment^24^. Previous gene expression studies in altered gravity have been mostly end-point measurements after time periods of hours or longer in microgravity and focused on the identification of particular gravity-responsive genes. Since gene expression responds very rapidly to altered gravity within or less than minutes^28,29^ and force transduction into chromatin requires only seconds^12^, initial mechanisms can be studied in the minute range, where different microgravity platforms (parabolic flights, suborbital ballistic rockets) are available for multi-platform analysis at an integrative level^4,29^.

Therefore, we recently investigated the dynamics of gene expression response to different gravitational environments in human Jurkat T lymphocytic cells during parabolic flight and suborbital rocket experiments^4,29^, identified gravity-regulated genes, but also revealed an overall high stability of gene expression in microgravity^4^. Experiments with cells of the immune system not only address fundamental biological questions about the effects of gravity on cellular homeostasis, but also the important medical risk of exploration class long-term manned space missions requiring mitigation^30^. Thus, the immune system belongs to the most affected systems during spaceflight (reviewed in)^31–33^ and sensitivity of cells of the human immune system to reduced gravity has been confirmed in numerous studies in real and simulated microgravity in T lymphocytes and cells of the monocyte-macrophage-system (MMS)^33–39^, but also indicated the existence of fast cellular adaptation^38^. In this study, we therefore focused on the first and initial transcriptome events in cells of the MMS. Due to the operational constraints of the conducted experiment missions, we used U937 human myelomonocytic cells, as established during other microgravity and space experiments^36,37^.

In addition to the hypothesis of direct force transduction into chromatin, force-sensitive ion channels have been discussed as trigger point of mechanotransduction^7,8,40,41^ into complex cellular reactions such as gene expression. Macrophages harbor ATP-gated P2X channels, store-operated Orai channels and members of the transient receptor potential (TRP) cation (TRPC) channel family, which play important roles in inflammation and phagocytosis^42–48^. Because TRPC1 is activated by stretch^49–51^, it represents a candidate for transduction of gravitational forces in cells of the MMS. This hypothesis was corroborated by the finding that in the unicellular photosynthetic flagellate *Euglena gracilis*, knockdown of a putative TRP channel abolished gravitaxis^52^. In order to test a potential role of ion channels and in particular TRPC channels, we also conducted inhibitor experiments with SKF-96365, commonly used to characterize the potential functions of TRPC channels and blocking voltage-activated calcium channels^53,54^, sarco- and endoplasmic reticula Ca^2+^ pumps^55,56^, voltage-gated sodium currents^57,58^, and ATP-sensitive and voltage-gated potassium channels^58,59^. Therefore, the combination of a wide range of ion channel inhibition with whole transcriptome analysis offers the possibility to study the functional role of ion channels in the transduction of gravitation forces at an integrative level.

Aim of this study was to investigate the transcriptome in force-free conditions of microgravity and in hypergravity, using different research platforms, gravity conditions and time points in order to identify potential molecular candidates of gravitational force-responsive gene regulation and to test the role of ion channels in gravitational force-dependent gene regulation. Our approach allowed the identification and validation of gravity-regulated gene expression through two fully independent large-scale research campaigns. Therefore, transcriptional changes identified after both campaigns are characterized by a high level of evidence due to independent sets of experiments in combination with independent research platforms.

## Results

**Figure 1:**
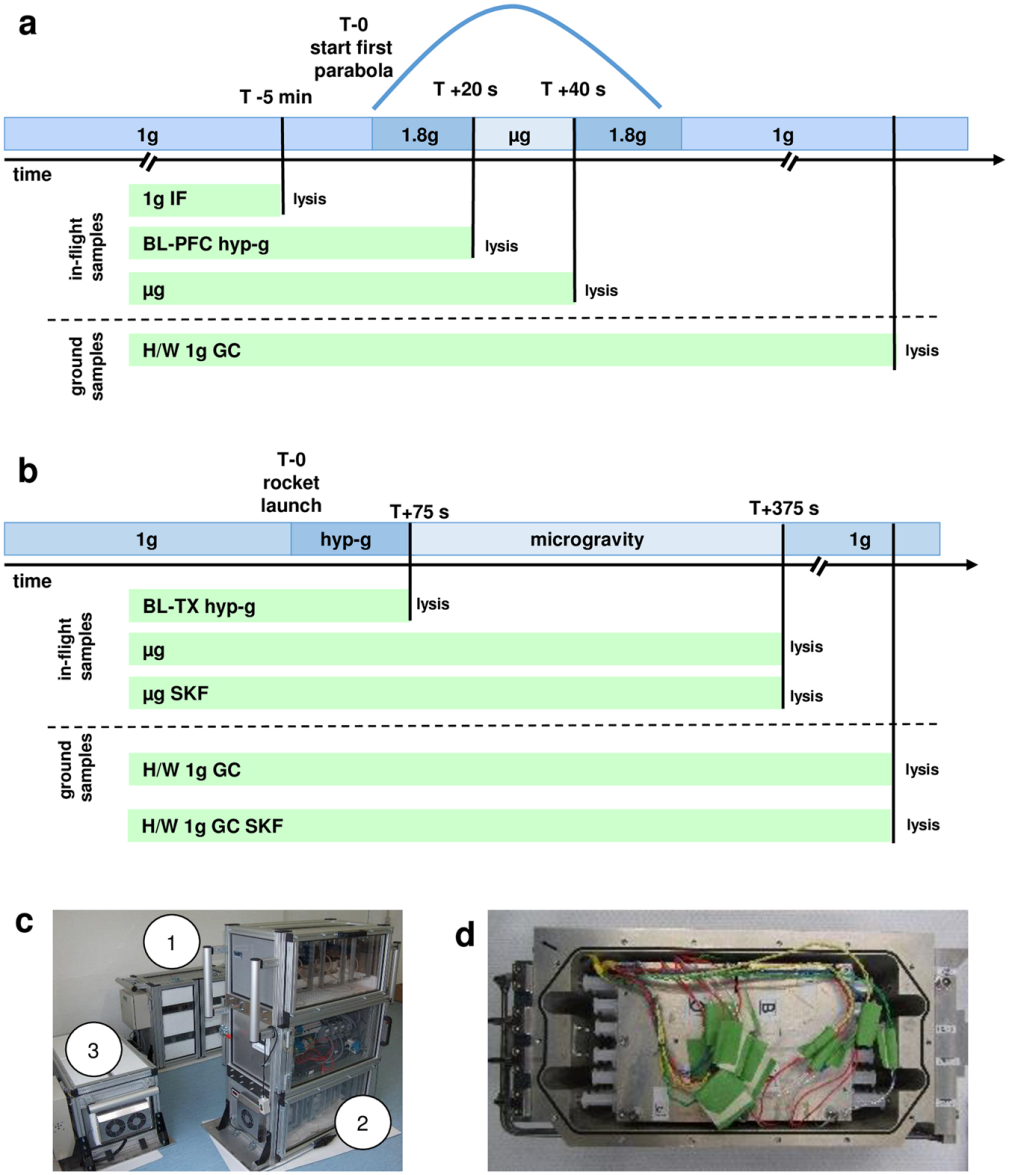
Sample lysis scheme. (**a**) U937 cells were analyzed during the 19^th^ DLR parabolic flight campaign. In total, four sample groups were lysed at defined g conditions and time points: (1) 1 g in-flight (1 g IF) 5 min before the first parabola, (2) 1.8 g samples at the end of the first parabola after 20 s of the 1.8 g hypergravity phase; these samples also serve as baseline (BL-PFC hyp-g) directly before the microgravity phase, (3) at the end of the first parabola after 20 s of microgravity (μg) phase, and (4) 1 g hardware ground controls (H/W 1 g GC), directly after the flight inside the aircraft. (**b**) U937 cells were investigated during the TEXUS-49 suborbital ballistic rocket campaign. Overall, five sample groups were lysed at set time points and g conditions: (1) the baseline (BL-TX hyp-g) group monitored the first 75 s of the flight after liftoff including hypergravity and vibrations, (2) microgravity samples (µg) were lysed 375 s post-launch, resulting in 300 s of microgravity time, (3) microgravity samples lysed 375 s post-launch with the cation ion channel inhibitor SKF-96365 (µg SKF), (4) 1 g hardware ground controls (H/W 1 g GC) lysed approximately 15 min after launch and (5) 1 g hardware ground controls with SKF-96365 (H/W 1 g GC SKF) lysed approximately 15 min after launch. (**c**) Experiment hardware of the parabolic flight (19^th^ DLR PFC). In-flight experiment system for parabolic flights on board the Airbus A300 ZERO-G. Experiment hardware structure which consists of an incubator rack to store the cell containers at 36.5 °C before the experiment (1), an experiment rack, in which all technical aggregates are accommodated for the execution of the experiment and where the living cells are processed during altered gravity (2), and a cooling rack to store all cell containers at 4 °C after the injection of the lysis solution until landing (3). (**d**) Experiment hardware of the suborbital ballistic rocket (TEXUS-49) experiments. TEXUS consists of a VSB-30 engine (not shown) and of the payload structure. Sets of three sterile syringes were filled with cell suspension, medium with or without SKF-96365, and lysis buffer connected by a T-piece with small plugs at the outlet ports to prevent premature contact of the fluids. The syringe systems are accommodated in tempered and vacuum-resistant containers (shown).

During the parabolic flight campaign (19^th^ DLR PFC), U937 cells were subjected to 20 s of hypergravity (1.8 g) and subsequently to 20 s of microgravity during the first parabola, and samples were obtained at the end of each flight phase. Control samples were obtained in-flight 5 min before the first parabola and on ground (Table 1, Fig. 1). In case of the TEXUS-49 suborbital rocket flight, samples were acquired 75 s after lift-off after the hypergravity launch phase and before the microgravity phase and after 5 min of microgravity flight. Control samples were prepared on ground. Further sample sets in microgravity and on ground were obtained in presence of 25 µM SKF-96365, an ion channel inhibitor of TRPC channels, voltage-gated Ca^2+^ channels and potassium channels. For both campaigns, RNA from at least four samples in each group was isolated, labeled and hybridized on a microarray chip.

**Table 1:** Nomenclature of experiment groups of the 19^th^ DLR parabolic flight and TEXUS-49 suborbital ballistic rocket campaigns.

		19^th^ DLR PFC	TEXUS-49
Gravity condition	Hardware 1 g ground control	H/W 1 g GC	H/W 1 g GC
	1 g in-flight	1 g IF	n/a
	Baseline/Hypergravity [directly before µg phase]	BL-PFC hyp-g [1.8 g, 20 s]	BL-TX hyp-g [max. 13.5 g, 75 s]
	Microgravity (µg)	µg [20 s]	µg [300 s]
	Hardware 1 g ground control with SKF	n/a	H/W 1 g GC SKF
	Microgravity (µg) with SKF	n/a	µg SKF [300 s]

Additional information on the group is given in squared brackets. SKF: SKF-96365, n/a: not applicable.

### Rapid and extensive transcriptome alterations after 20 s of altered gravity

During the first parabola in the parabolic flight experiment, the transcriptome of human U937 cells responded rapidly within 20 s of hypergravity with a total number of 17998 (17228 annotated) (BL-PFC hyp-g versus H/W 1 g GC) differentially regulated transcripts. After the subsequent first microgravity phase, 11810 (11361 annotated) transcripts (µg versus H/W 1 g GC) were differentially expressed, which is 2.7-fold the number of 4293 (4165 annotated) transcripts that were altered solely as a result of the flight conditions without altered gravity (comparison 1 g IF versus H/W 1 g GC, Table 2).

**Table 2:** Numbers of differentially expressed transcripts in comparisons of the experiment groups of the 19^th^ DLR parabolic flight campaign.

		1 g IF vs H/W 1 g GC	BL-PFC hyp-g vs 1 g IF	µg vs BL-PFC hyp-g	µg vs 1 g IF	BL-PFC hyp-g vs H/W 1 g GC	µg vs H/W 1 g GC
differentially expressed transcripts	up-regulated	748	6564	709	494	8170	3089
	down-regulated	3545	5118	1102	1220	9828	8721
	**total number**	**4293**	**11682**	**1811**	**1714**	**17998**	**11810**
differentially expressed annotated transcripts	up-regulated	707	6159	669	457	7635	2852
	down-regulated	3458	4981	1046	1196	9593	8509
	**total number**	**4165**	**11140**	**1715**	**1653**	**17228**	**11361**

Differential expression is defined as t-test p-value < 0.05; fold change ≤−1.3 or ≥ +1.3. The term “transcripts” refers to all 45034 transcripts of the array, whereas “annotated transcripts” refers to the 42947 transcripts that are annotated to a gene.

**Figure 2:**
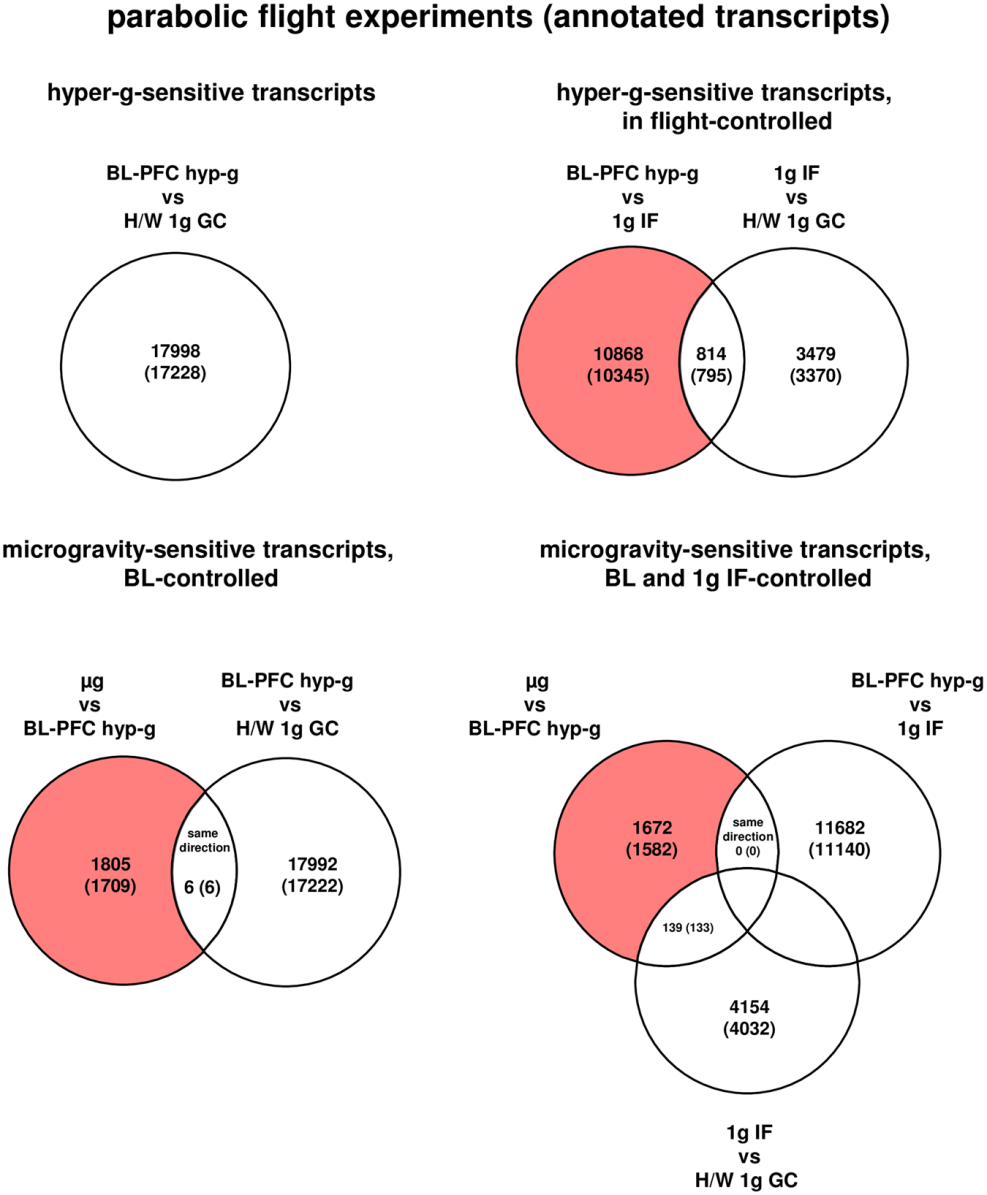
Differentially regulated transcripts (and annotated transcripts) in human U937 myelomonocytic cells during the 19^th^ DLR Parabolic Flight Campaign. The different comparisons and resulting intersections for hypergravity and microgravity-sensitive transcripts are displayed. Fold change ≤−1.3 and ≥+1.3, p < 0.05.

After exclusion of all transcripts already altered due to flight conditions (comparison 1 g IF versus H/W 1 g GC), we revealed 10345 hypergravity-sensitive annotated transcripts. In the microgravity-phase (µg versus BL-PFC hyp-g) we identified 1715 microgravity-sensitive annotated transcripts (Table 2, Table 3, and Fig. 2). Six transcripts were differentially expressed in the same direction in the microgravity phase as in the hypergravity phase and were therefore eliminated in order to exclude potential protracted effects, resulting in 1709 differentially regulated transcripts (Table 3 and Fig. 2). The expression fold change (FC) for the hypergravity-sensitive transcripts was in the range between +4.68 (up-regulation) and −7.8 (down-regulation) with average values of +1.97 and −1.79, respectively. The expression fold change (FC) for the microgravity-sensitive transcripts was in the range between +3.35 (up-regulation) and −2.66 (down-regulation) with average values of +1.52 and −1.58, respectively (Table 3).

**Table 3:** Numbers of transcripts that are differentially expressed in response to altered gravitational conditions in the 19^th^ DLR parabolic flight campaign.

		**hypergravity-sensitive transcripts** differentially expressed in BL-PFC hyp-g vs H/W 1 g GC (TEXUS-49-analogous)	average FC of BL-PFC hyp-g vs H/W 1 g GC	min/max FC of BL-PFC hyp-g vs H/W 1 g GC	**hypergravity-sensitive transcripts**, **in flight-controlled** differentially expressed in BL-PFC hyp-g vs 1 g IF, but not differentially expressed in 1 g IF vs H/W 1 g GC	average FC of BL-PFC hyp-g vs 1 g IF	min/max FC of BL-PFC hyp-g vs 1 g IF	**microgravity-senstitive transcripts**, **BL-controlled** differentially expressed in µg vs BL-PFC hyp-g, but not differentially expressed in the same direction in BL-PFC hyp-g vs H/W 1 g GC (TEXUS-49-analogous)	average FC of µg vs BL-PFC hyp-g	min/max FC of µg vs BL-PFC hyp-g	**microgravity-senstitive transcripts**, **BL and 1 g IF-controlled** differentially expressed in µg vs BL-PFC hyp-g, but not differentially expressed in 1 g IF vs H/W 1 g GC or in the same direction in BL-PFC hyp-g vs 1 g IF	average FC µg vs BL-PFC hyp-g	min/max µg vs BL-PFC hyp-g
differentially expressed transcripts	up-regulated	8170	1.971	4.681	6423	1.981	4.681	708	1.518	3.345	629	1.519	3.345
	down-regulated	9828	−1.791	−7.802	4445	−1.794	−7.802	1097	−1.579	−2.659	1043	−1.585	−2.659
	**total number**	**17998**			**10868**			**1805**			**1672**		
differentially expressed annotated transcripts	up-regulated	7635	1.974	4.681	6023	1.983	4.681	668	1.516	3.345	592	1.516	3.345
	down-regulated	9593	−1.790	−7.802	4322	−1.794	−7.802	1041	−1.581	−2.659	990	−1.586	−2.659
	**total number**	**17228**			**10345**			**1709**			**1582**		

Transcripts which were significantly changed in the control comparisons were eliminated. Differential expression is defined as t-test p-value < 0.05; fold change (FC) ≤ −1.3 or ≥ +1.3. FCs are ratios between the averages of linear expression values. If the ratio is <1, FC is calculated as the negative reciprocal of the ratio.

### Extensive transcriptome alterations after 75 s of hypergravity and 300 s of microgravity

**Figure 3:**
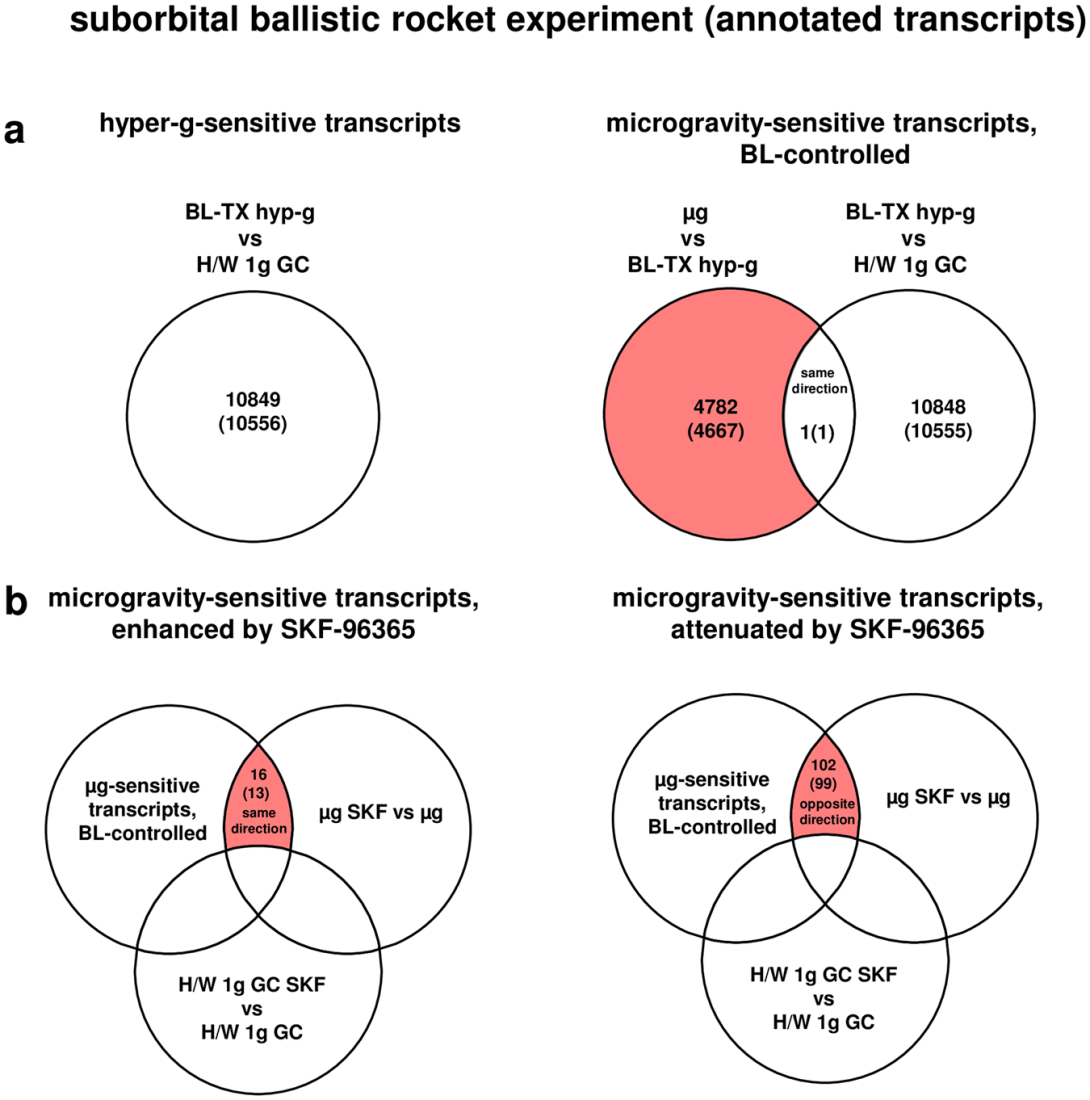
Differentially regulated transcripts (and annotated transcripts) in human U937 myelomonocytic cells during the TEXUS-49 suborbital ballistic rocket campaign. (**a**) The different comparisons and resulting intersections for hypergravity and microgravity-sensitive transcripts are displayed. (**b**) Analysis of the influence of the cation ion channel blocker SKF-96365 on microgravity-regulated gene expression. Fold change ≤−1.3 and ≥+1.3, p < 0.05.

During the launch phase of the suborbital ballistic rocket experiment, the transcriptome of human U937 cells responded rapidly within 75 s covering the hypergravity phase with a total number of 10849 (10556 annotated) differentially regulated transcripts (comparison BL-TX hyp-g versus H/W 1 g GC, Table 4, Fig. 3). After the subsequent 300 s microgravity phase, 4783 (4668 annotated) transcripts were differentially expressed (µg vs BL-TX hyp-g, Table 4), which is 2.3-fold lower than the number of differentially expressed transcripts in hypergravity. 1291 (1247 annotated) transcripts were differentially expressed in microgravity when compared to the H/W 1 g ground control group (Table 4). One single transcript was differentially expressed in the same direction in hypergravity (BL-TX hyp-g vs H/W 1 g GC) and microgravity (µg vs BL-TX hyp-g) and was therefore eliminated in order to exclude potential protracted effects, resulting in 4667 microgravity-sensitive baseline (BL)-controlled annotated transcripts (Table 5 and Fig. 3a). The expression fold change (FC) was in the range between +8.85 (up-regulation) and -3.38 (down-regulation) with average values of +2.11 and −1.68 respectively for the hypergravity-sensitive transcripts and between +7.23 (up-regulation) and −3.26 (down-regulation) with average values of +1.85 and −1.71 respectively for microgravity-sensitive transcripts (Table 5).

**Table 4:** Numbers of differentially expressed transcripts in comparisons of the experiment groups of TEXUS-49 suborbital ballistic rocket campaign.

		µg vs BL-TX hyp-g	BL-TX hyp-g vs H/W 1 g GC	µg vs H/W 1 g GC
differentially expressed transcripts	up-regulated	888	8309	300
	down-regulated	3895	2540	991
	**total number**	**4783**	**10849**	**1291**
differentially expressed annotated transcripts	up-regulated	826	8195	286
	down-regulated	3842	2361	961
	**total number**	**4668**	**10556**	**1247**

Differential expression is defined as t-test p-value < 0.05; fold change ≤ −1.3 or ≥+1.3. The term “transcripts” refers to all 45034 transcripts of the array, whereas “annotated transcripts” refers to the 42947 transcripts that are annotated to a gene.

**Table 5:** Numbers of transcripts that are differentially expressed in response to altered gravitational conditions in the TEXUS-49 suborbital ballistic rocket campaign.

		hypergravity-sensitive transcripts differentially expressed in BL-TX hyp-g vs H/W 1 g GC	average FC BL-TX hyp-g vs H/W 1 g GC	min/max FC BL-TX hyp-g vs H/W 1 g GC	microgravity-sensitive transcripts, BL-controlled differentially expressed in µg vs BL-TX hyp-g, but not differentially expressed in the same direction in BL-TX hyp-g vs H/W 1 g GC	average FC µg vs BL-TX hyp-g	min/max FC µg vs BL-TX hyp-g
differentially expressed transcripts	up-regulated	8309	2.109	8.848	888	1.845	7.225
	down-regulated	2540	−1.676	−3.383	3894	−1.712	−3.259
	**total number**	**10849**			**4782**		
differentially expressed annotated transcripts	up-regulated	8195	2.109	8.848	826	1.850	7.225
	down-regulated	2361	−1.669	−3.383	3841	−1.713	−3.259
	**total number**	**10556**			**4667**		

Transcripts which were significantly changed in the control comparisons were eliminated. Differential expression is defined as t-test p-value < 0.05; fold change (FC) ≤ −1.3 or ≥+1.3. FCs are ratios between the averages of linear expression values. If the ratio is <1, FC is calculated as the negative reciprocal of the ratio.

### Role of SKF-96365-sensitive ion channels in gravity-regulated gene expression

Because force-sensitive ion channels have been discussed to induce transduction of mechanical forces into complex cellular reactions^7,8,40,41^, we used the wide-range ion channel inhibitor SKF-96365 to identify ion channel-dependent regulated gene expression in altered gravity. The number of differentially expressed transcripts with and without incubation with SKF-96365 are shown in Table 6 (comparisons H/W 1 g GC SKF versus H/W 1 g GC and µg SKF versus H/W 1 g GC SKF). SKF-96365 had distinct effects on gene expression: 4920 (4732 annotated) transcripts were differentially regulated in 1 g. In microgravity, SKF-96365 more than doubled the number of differentially regulated genes to 10959 (10413 annotated) transcripts (Table 6). Differentially regulated transcripts were now further verified by the appropriate control experiments (Table 7) and only transcripts were selected, which were (1) microgravity-sensitive (baseline controlled), (2) differentially expressed in the comparison µg SKF versus µg either in the same direction (enhancement of microgravity effect) or in the opposite direction (reversal of microgravity effect), and (3) that were not differentially expressed in 1 g (comparison H/W 1 g GC SKF-96365 versus H/W 1 g GC). In total, 13 annotated transcripts showed an enhanced sensitivity to microgravity and 99 annotated transcripts showed a decreased sensitivity to microgravity in the presence of SKF-96365 (Table 7, Supplementary Table 1, and Fig. 3b). The expression fold change (FC) was in the range between +1.8 (up-regulation) and −1.43 (down-regulation) with average values of +1.5 and −1.43 respectively for annotated transcripts with an enhanced microgravity sensitivity and between +2.04 (up-regulation) and −4.17 (down-regulation) with average values of +1.5 and −1.89 respectively for annotated transcripts with an attenuated microgravity sensitivity (Table 7). In conclusion, we could attribute only 118 (112 annotated) out of 4782 (4667 annotated) microgravity-regulated transcripts, equivalent to 2.4%, to gravity-induced functional effects of SKF-96365-sensitive ion channels.

**Table 6:** Numbers of transcripts that are differentially expressed in comparisons of the experiment groups of the TEXUS-49 suborbital ballistic rocket campaign containing the ion channel blocker SKF-96365.

		H/W 1 g GC SKF vs H/W 1 g GC	µg SKF vs H/W 1 g GC SKF
differentially expressed transcripts	up-regulated	3876	5013
	down-regulated	1044	5946
	**total number**	**4920**	**10959**
differentially expressed annotated transcripts	up-regulated	3764	4715
	down-regulated	968	5698
	**total number**	**4732**	**10413**

Differential expression is defined as t-test p-value < 0.05; fold change ≤−1.3 or ≥+1.3. The arrays comprise 45034 transcripts of which 42947 are annotated to a gene.

**Table 7:** Numbers of transcripts that are differentially expressed in response to altered gravitational conditions in an SKF-96365-sensitive manner in the TEXUS-49 suborbital ballistic rocket campaign.

		microgravity effect is enhanced by ion-channel inhibition with SKF	average FC µg SKF vs µg	max/min FC µg SKF vs µg	microgravity effect is attenuated by ion-channel inhibition with SKF	average FC µg SKF vs µg	max/min FC µg SKF vs µg
differentially expressed transcripts	up-regulated	14	1.483	1.803	48	−1.871	−4.167
	down-regulated	2	−1.373	−1.429	54	1.494	2.038
	**total number**	**16**			**102**		
differentially expressed annotated transcripts	up-regulated	12	1.496	1.803	46	−1.889	−4.167
	down-regulated	1	−1.429	−1.429	53	1.498	2.038
	**total number**	**13**			**99**		

Groups comprise transcripts that are 1.) microgravity-sensitive, BL-controlled, 2.) differentially expressed in the comparison µg SKF vs µg either in the same direction (enhancement) or in the opposite direction (attenuation), and 3.) not differentially expressed in the comparison H/W 1 g GC SKF vs H/W 1 g GC (exclusion of transcripts that are sensitive to SKF). Differential expression is defined as t-test p-value < 0.05; fold change (FC) ≤−1.3 or ≥+1.3. FCs are ratios between the averages of linear expression values. If the ratio is <1, FC is calculated as the negative reciprocal of the ratio.

### Gene ontology enrichment analyses

We performed Gene Ontology (GO) enrichment analyses for an integrative evaluation of the effects of altered gravity on cellular processes and functions. 20 s of microgravity influenced catabolic processes and cation channel transcripts, whereas 20 s of hypergravity had an impact on DNA replication and microtubule-based processes (Supplementary Figs S1 and S2). 75 s of hypergravity and 300 s of microgravity generated a higher number of affected cellular processes and functions with partially overlapping aspects (Supplementary Figs S3 and S4). 300 s of microgravity influenced transcripts for intracellular transport, RNA and enzyme binding, mRNA processing, posttranscriptional regulation of gene expression, cell cycle and cell division (Supplementary Fig. S3). 75 s spanning hypergravity impacts transcripts of metabolic processes, regulation of transcription, intracellular transport, cell cycle and cell division (Supplementary Fig. S4).

### No strong effects on apoptosis/necroptosis-associated pathways in altered gravity

In order to investigate proposed effects of altered gravity on apoptosis^60,61^, we screened our data sets for genes involved in apoptosis and necroptosis and identified 553 genes from KEGG pathways (Apoptosis - Homo sapiens PATHWAY: hsa04210; Necroptosis - Homo sapiens PATHWAY: hsa04217) and the review from Elmore^62^ and found that apoptosis/necroptosis-associated pathways are only slightly or not altered at all in microgravity (Supplementary Tables S2–S5).

### Transcripts were primarily upregulated in hypergravity and down-regulated in microgravity

**Figure 4:**
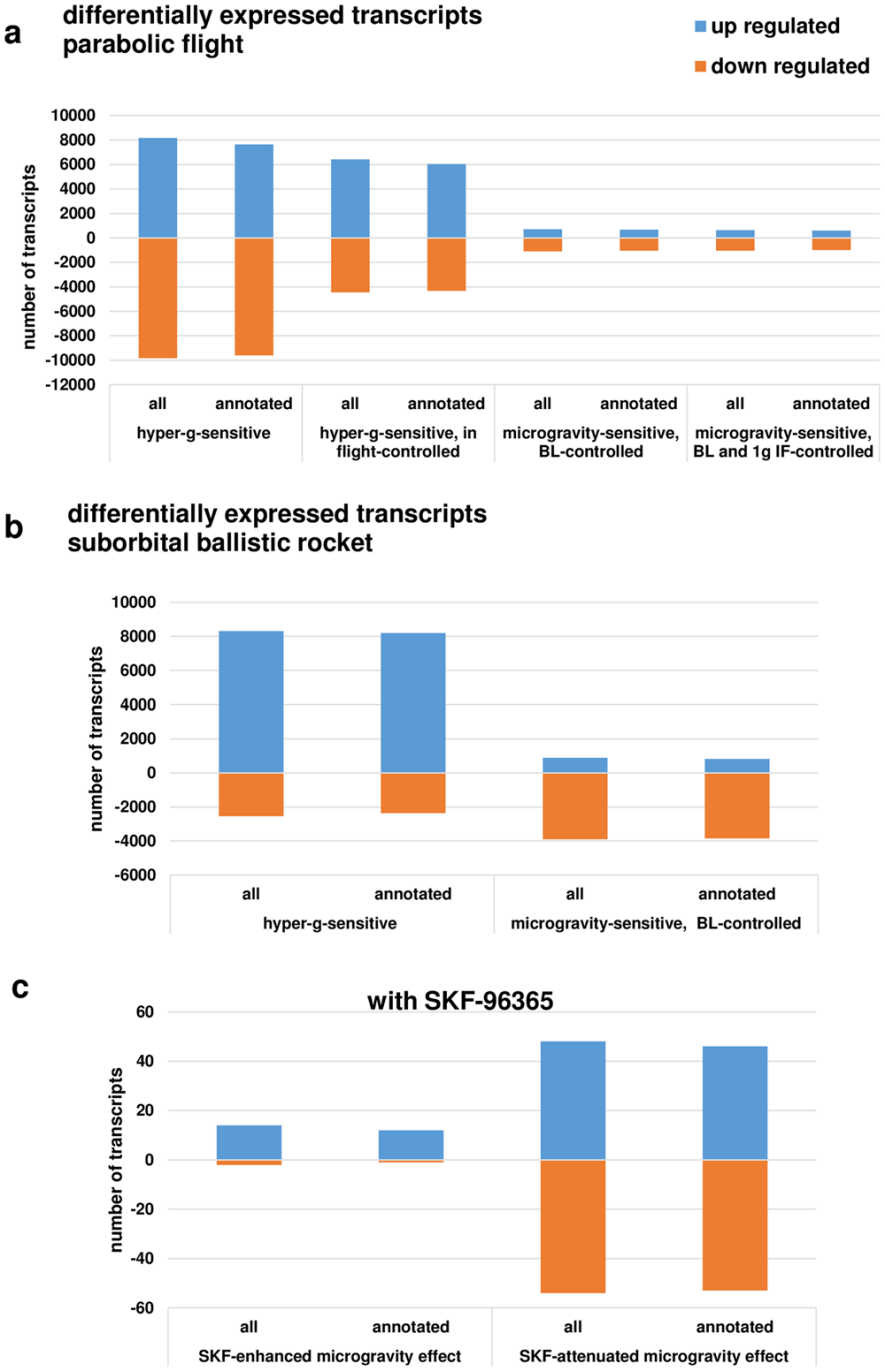
Distribution of differentially expressed transcripts. (**a**) Hyper- and microgravity-sensitive transcripts identified for the 19^th^ DLR Parabolic Flight Campaign. (**b**) Hyper- and microgravity sensitive transcripts identified for the TEXUS-49 suborbital ballistic rocket campaign, (**c**). SKF-96365-dependent hyper- and microgravity-sensitive transcripts.

Figure 4 summarizes the number of differentially expressed genes in the parabolic flight and suborbital ballistic rocket experiments. Whereas up- or down-regulation was almost equally distributed in differentially expressed transcripts after 20 s of altered gravity (Fig. 4a), longer periods of hypergravity resulted in primarily up-regulation of transcripts and longer periods of microgravity in down-regulation of transcripts (Fig. 4b). In the case of the ion channel inhibitor SKF-96365 enhancing the effect of microgravity, transcripts were primarily up-regulated, in the case of SKF-96365 reversed the effect of microgravity, transcripts were almost equally distributed between up- and down-regulation (Fig. 4c).

### Evidence for rapid counter-regulation of initially altered transcripts

**Figure 5:**
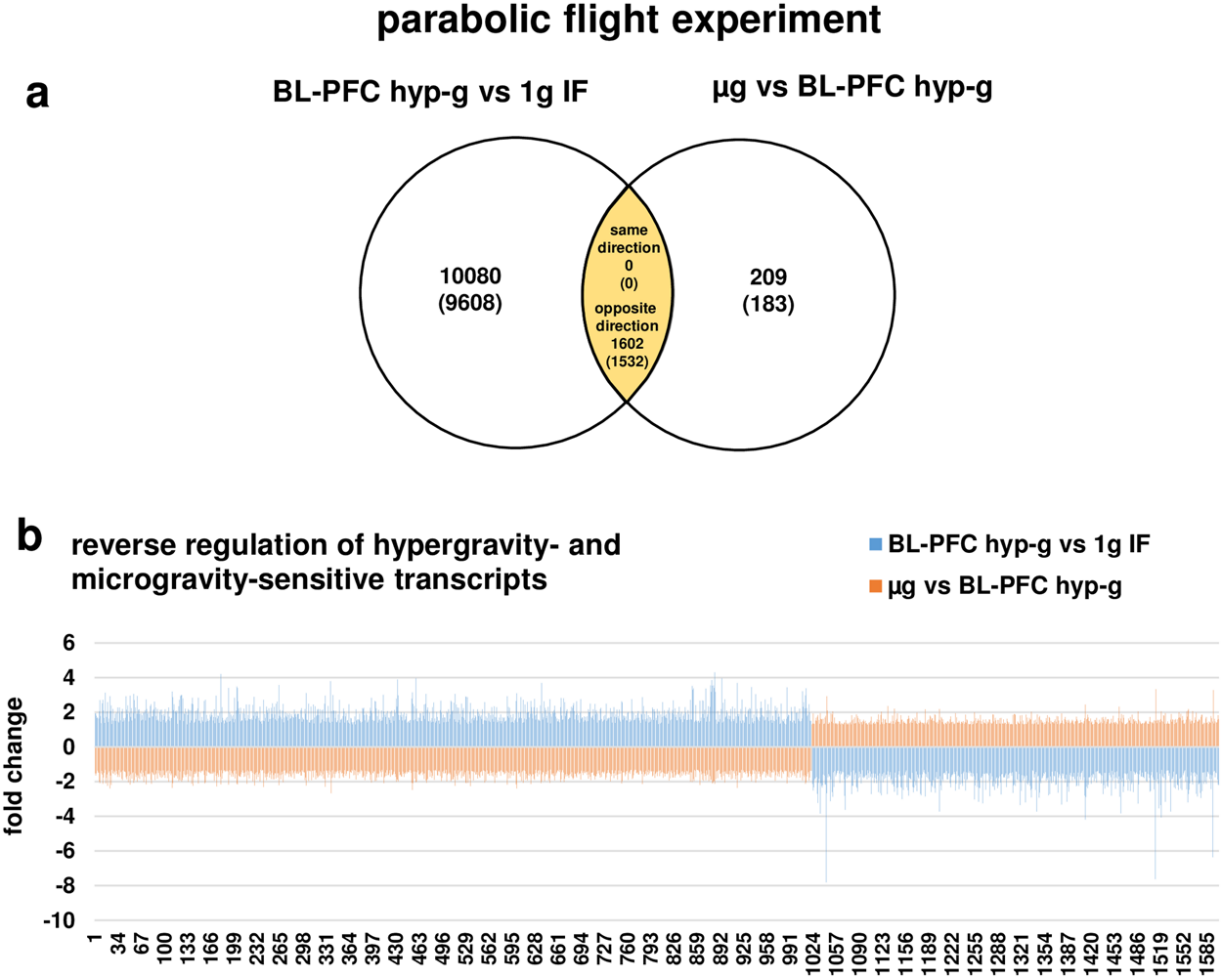
Hyper- and microgravity-double-sensitive transcripts were reversely regulated in the 19^th^ DLR Parabolic Flight Campaign. (**a**) Venn diagram showing 1602 hyper- and microgravity-double-sensitive transcripts (1532 annotated transcripts). (**b**) Graphical display of the 1602 transcripts of the intersection. Fold changes (FCs) are ratios between the averages of linear expression values. If the ratio is <1, FC is calculated as the negative reciprocal of the ratio.

**Figure 6:**
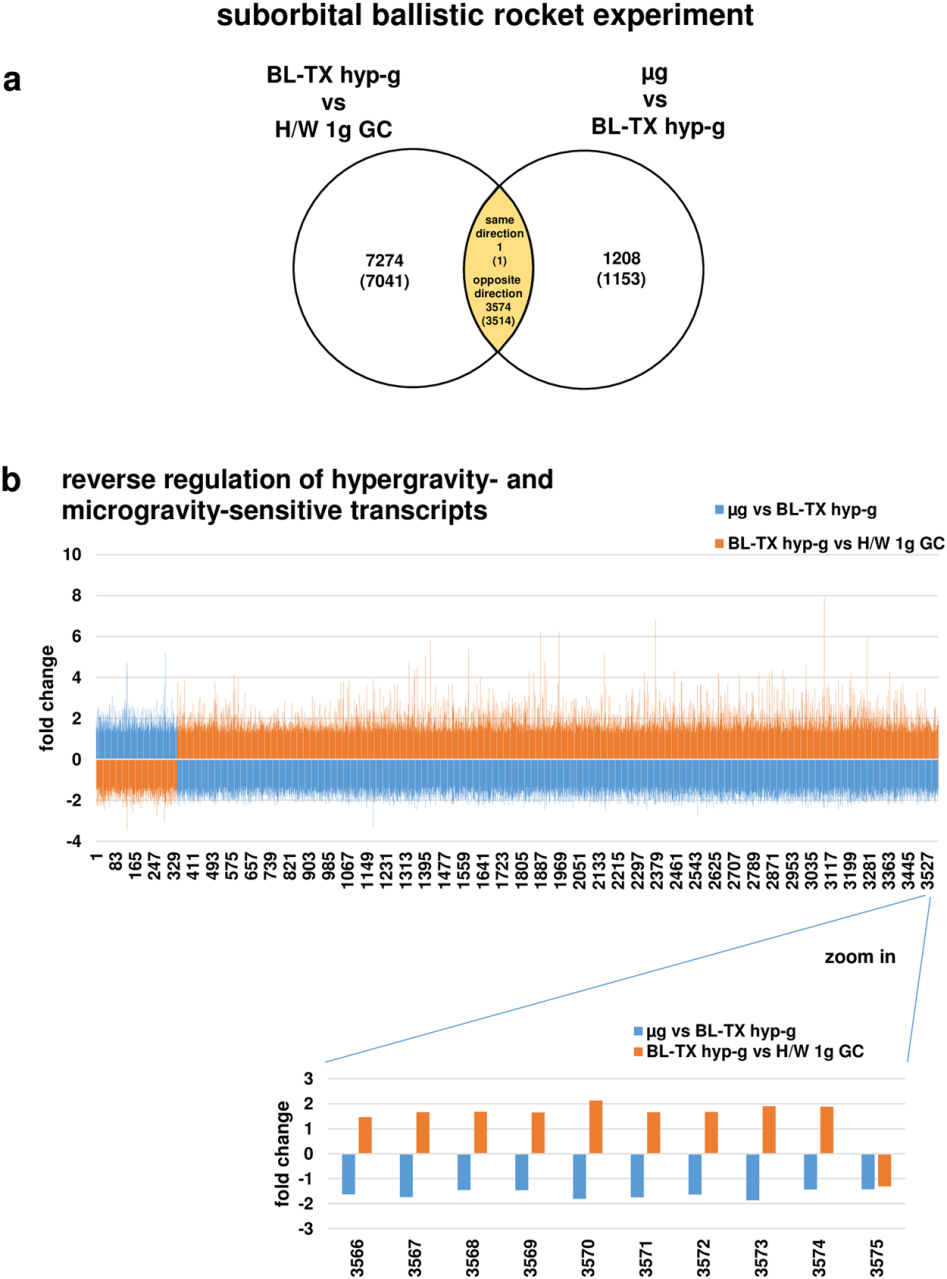
Hyper- and microgravity-double-sensitive transcripts are reversely regulated in the TEXUS-49 suborbital ballistic rocket campaign. (**a**) Venn diagram showing 3575 hyper- and microgravity-double-sensitive transcripts (3515 annotated transcripts). (**b**) Graphical display of 3575 transcripts of the intersection, including one single transcript that is regulated in the same direction. Fold changes (FCs) are ratios between the averages of linear expression values. If the ratio is <1, FC is calculated as the negative reciprocal of the ratio.

**Figure 7:**
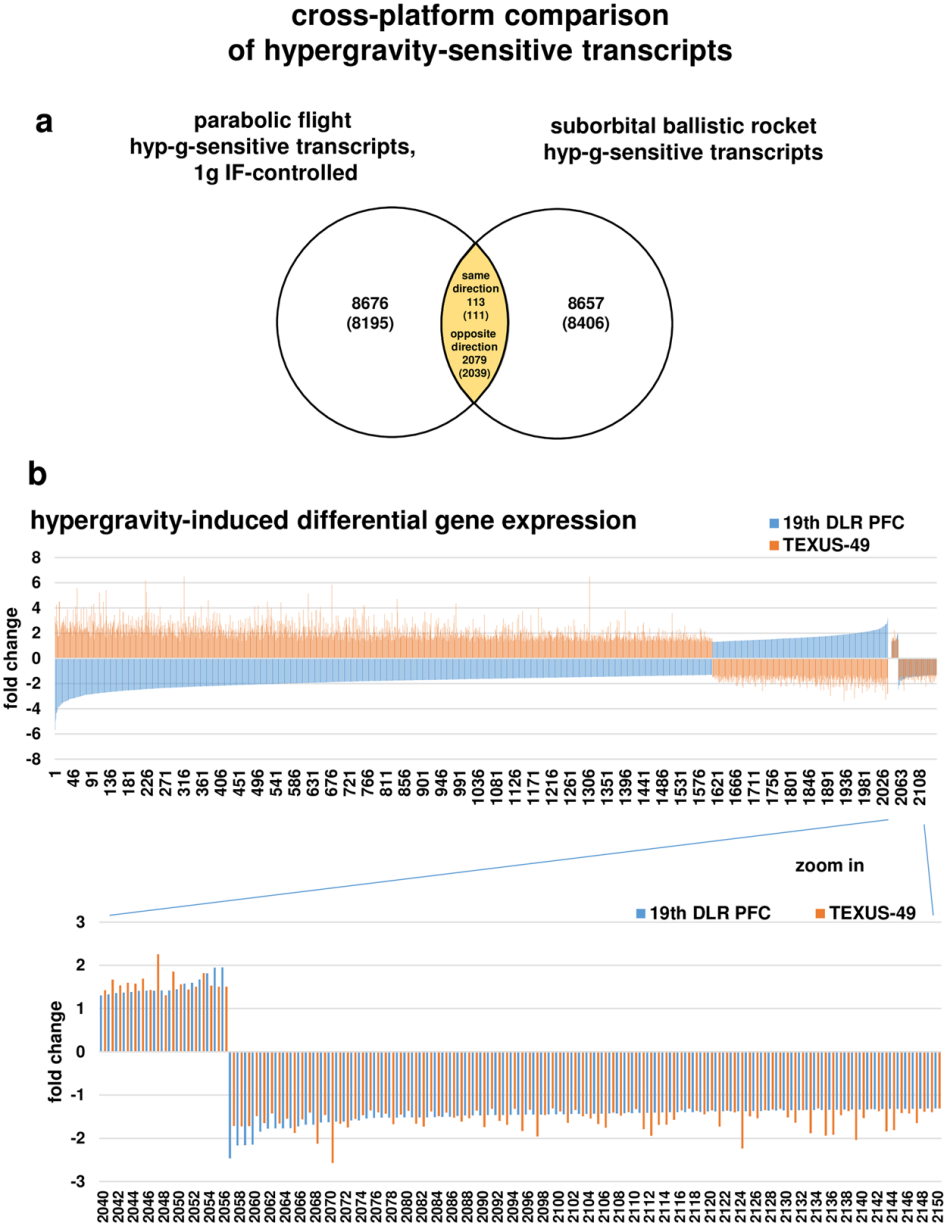
Hypergravity-sensitive transcripts are reversely regulated when comparing 20 s (19^th^ DLR Parabolic Flight Campaign) and 75 s (TEXUS-49 suborbital ballistic rocket campaign) exposure times. (**a**) Venn diagram showing the intersection of hypergravity-sensitive transcripts of both platforms. (**b**) Graphical display of transcripts (annotated transcripts) showing that transcripts are mostly regulated in the opposite direction. Only 113 from 2192 transcripts were regulated in the same direction. Fold changes (FCs) are ratios between the averages of linear expression values. If the ratio is <1, FC is calculated as the negative reciprocal of the ratio.

**Figure 8:**
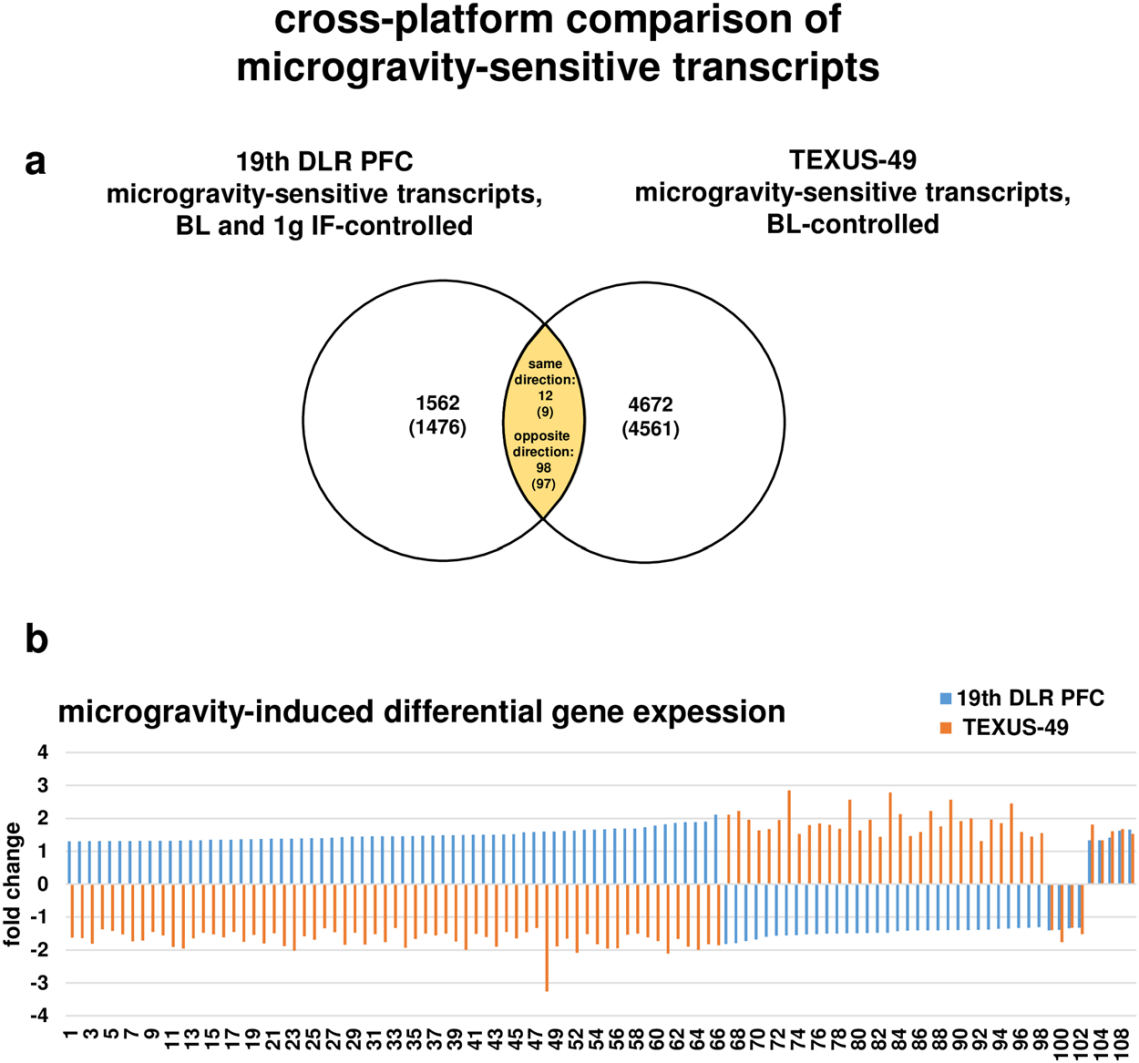
Microgravity sensitive transcripts are reversely regulated when comparing 20 s (19^th^ DLR Parabolic Flight Campaign) and 300 s (TEXUS-49 suborbital ballistic rocket campaign) exposure times. (**a**) Venn diagram showing the intersection of microgravity-sensitive transcripts of both platforms. (**b**) Graphical display of transcripts (annotated transcripts) showing that transcripts are mostly regulated in the opposite direction. Only 12 out of 110 transcripts are regulated in the same direction. Fold changes (FCs) are ratios between the averages of linear expression values. If the ratio is <1, FC is calculated as the negative reciprocal of the ratio.

We then analyzed all transcripts from the parabolic flight experiments that were differentially expressed in both micro- and hypergravity, and identified 1602 (1532 annotated) transcripts (Fig. 5). Surprisingly, all identified transcripts were regulated in the opposite direction in hyper- and microgravity: Transcripts that were up-regulated in hypergravity, were down-regulated in microgravity and vice versa. None of the identified transcripts was regulated in the same direction in hypergravity and in microgravity (Fig. 5). We continued this analysis with the data from the suborbital ballistic rocket experiment and identified 3574 (3514 annotated) transcripts (Fig. 6) that were differentially expressed in hypergravity and in microgravity and revealed the same observation: All transcripts, except for one, were expressed also in the opposite direction in hyper- and microgravity, respectively (Fig. 6). Due to the nature of the flight profiles, the microgravity phase always followed the hypergravity phase in both flight experiments. We therefore investigated the hypothesis that reversely regulated transcripts are caused by counter-regulatory mechanisms. For this reason, we performed a cross-platform comparison of the hypergravity and microgravity phases, separately and with regard to their different duration. We identified 2192 (2150 annotated) transcripts that were altered in both platforms after 20 s and 75 s of hypergravity, respectively. We found that 2079 (2039 annotated) of these transcripts were regulated in the opposite direction, while 113 (111 annotated) transcripts were differentially expressed in the same direction (Fig. 7). The cross-platform comparison of the microgravity phases revealed 110 (106 annotated) transcripts that were altered in both platforms after 20 s and 300 s of microgravity, respectively. We found that 98 (97 annotated) transcripts were regulated in the opposite direction, while 12 (9 annotated) transcripts were differentially expressed in the same direction (Fig. 8). That means that more than 92% of microgravity-sensitive annotated transcripts that were differentially expressed on both platforms were regulated in opposite directions and are therefore adapting to the new gravitational environment by counter-regulation.

### Identification of gravity-regulated genes

**Figure 9:**
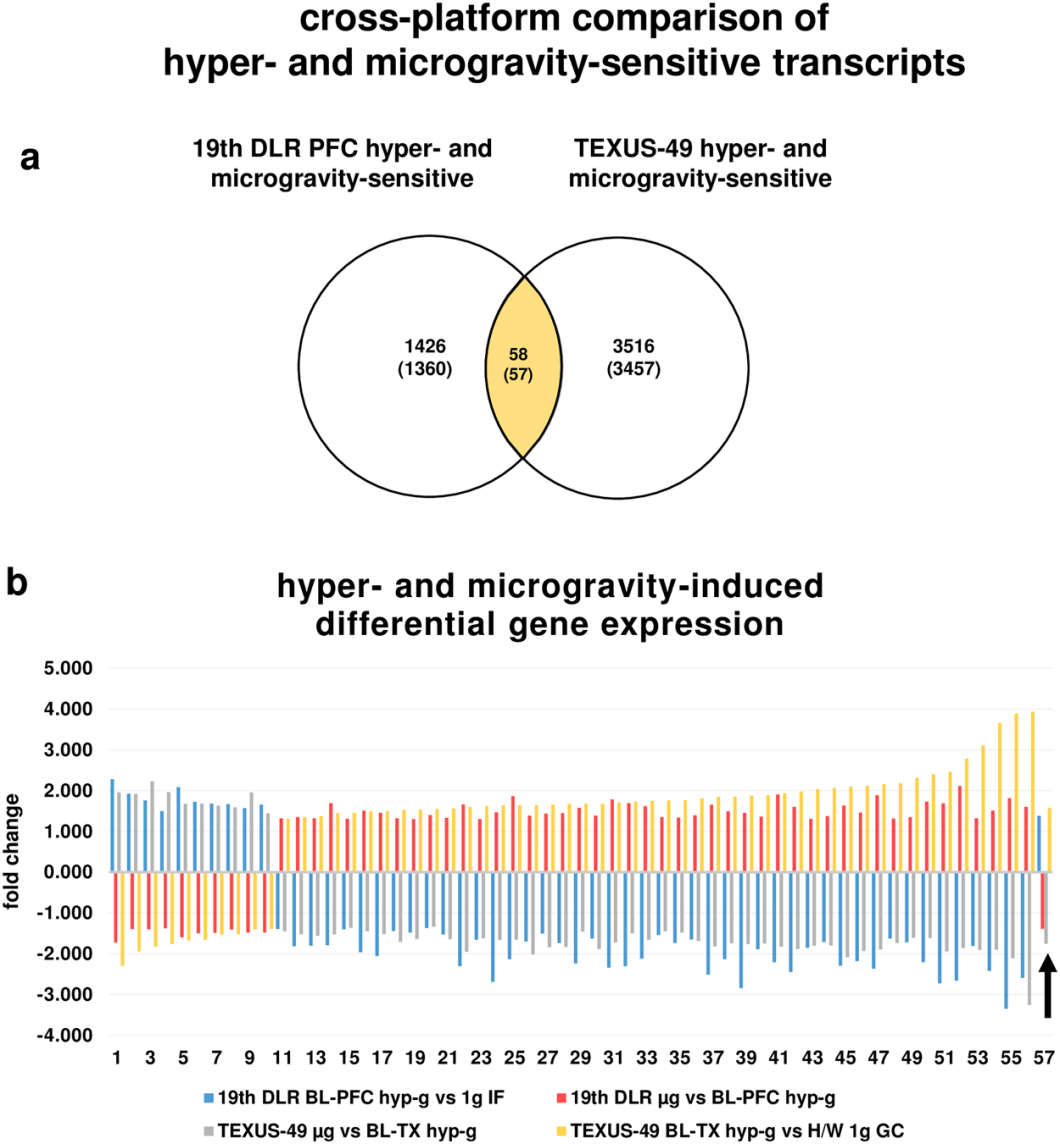
Hyper- and microgravity-double-sensitive transcripts identified for the 19^th^ DLR Parabolic Flight Campaign and the TEXUS-49 suborbital ballistic rocket campaign. (**a**) Venn diagram showing the intersection of hyper- and microgravity-double-sensitive transcripts of both platforms. (**b**) Graphical display of transcripts (annotated transcripts) showing that transcripts are regulated in the opposite direction between altered gravity conditions within one platform and between platforms. Only one transcript (arrow) was regulated in the same direction in both platforms. Fold changes (FCs) are ratios between the averages of linear expression values. If the ratio is <1, FC is calculated as the negative reciprocal of the ratio.

Inter-platform comparisons of all transcripts which were differently regulated in hyper- and microgravity revealed 58 (57 annotated) transcripts. All of these transcripts were reversely regulated between the two gravity conditions within one platform. Additionally, all transcripts but one were reversely regulated in the same gravity condition comparing both platforms (Fig. 9, Table 8). Interestingly, one of these 57 transcripts was ATP6V1E1, representing a vacuolar H^+^-ATPase (V-ATPase), already identified previously as a gravity regulated gene in human Jurkat T cells^29^.

**Table 8:** Differentially regulated annotated transcripts identified for the parabolic flight and sounding rocket campaign data sets.

gene name	probeset ID	19^th^ DLR PFC fold change		TEXUS-49 fold change	
		BL-PFC hyp-g vs 1 g IF	µg vs BL-PFC hyp-g	µg vs BL-TX hyp-g	BL-TX hyp-g vs H/W 1g GC
CAV1	AF172085	2.280	−1.727	1.955	−2.294
SEC14L3	BC069641	1.921	−1.393	1.914	−1.945
LOC646156	XM_933449	1.756	−1.403	2.228	−1.827
LRRN5	NM_006338	1.494	−1.375	1.961	−1.763
BCAM	BC050450	2.084	−1.594	1.673	−1.674
SPATA21	BC022039	1.722	−1.499	1.681	−1.657
ACRBP	NM_032489	1.676	−1.488	1.631	−1.530
FHAD1	XM_934885	1.667	−1.405	1.587	−1.522
RNF43	NM_017763	1.570	−1.480	1.956	−1.409
GPR45	NM_007227	1.653	−1.478	1.438	−1.389
CCDC25	NM_001031708	−1.395	1.318	−1.450	1.303
UVRAG	BC064837	−1.816	1.346	−1.526	1.351
MLSTD1	NM_018099	−1.802	1.319	−1.557	1.372
ATP6V1E1	NM_001039366	−1.789	1.688	−1.528	1.443
RPL4	NM_000968	−1.398	1.305	−1.366	1.453
ALKBH3	NM_139178	−1.966	1.508	−1.447	1.495
SKP2	NM_005983	−2.057	1.454	−1.515	1.497
ANAPC1	BC104902	−1.444	1.318	−1.707	1.524
KLHL12	NM_021633	−1.482	1.302	−1.638	1.530
LOC158345	XM_931828	−1.370	1.399	−1.339	1.543
C6orf120	NM_001029863	−1.531	1.333	−1.644	1.562
LOC643980	XM_928714	1.382	−1.386	−1.757	1.576
PFAAP5	BC010643	−2.302	1.663	−1.951	1.593
MRS2L	BC069009	−1.655	1.301	−1.623	1.618
RABGAP1L	AB019489	−2.690	1.464	−1.663	1.636
CLEC12A	BC063424	−2.134	1.863	−1.659	1.640
COX11	BC005895	−1.698	1.383	−2.020	1.641
RNF146	AK027558	−1.504	1.433	−1.840	1.656
SMC2L1	NM_006444	−1.736	1.448	−1.832	1.670
CACNA2D3	AF516696	−2.237	1.575	−1.457	1.679
SLFN11	BC052586	−1.628	1.382	−1.882	1.681
NUP88	NM_002532	−2.345	1.780	−1.722	1.701
CSTF3	NM_001033506	−2.306	1.691	−1.501	1.730
DKFZP686M0199	XM_932404	−2.119	1.619	−1.655	1.747
NUP205	NM_015135	−1.542	1.349	−1.450	1.757
XRN1	NM_019001	−1.737	1.333	−1.478	1.764
MASA	NM_021204	−1.651	1.394	−1.688	1.807
CCDC52	BC036951	−2.512	1.655	−1.821	1.842
VLDLR	NM_003383	−2.133	1.488	−1.740	1.849
LOC653198	XM_926455	−2.844	1.454	−1.759	1.876
C18orf25	NM_001008239	−1.888	1.361	−1.748	1.889
C11orf57	BC005403	−2.209	1.904	−1.827	1.936
LOC644305	XM_927477	−2.447	1.598	−1.886	1.973
PXMP3	BC005375	−1.856	1.305	−1.804	2.033
ZNF25	NM_145011	−1.713	1.372	−1.795	2.062
HEATR3	NM_017939	−2.295	1.627	−2.083	2.093
C14orf135	NM_022495	−2.186	1.455	−1.932	2.112
SLC35A1	AJ851889	−2.364	1.884	−1.897	2.162
SCYL2	NM_017988	−1.629	1.312	−1.737	2.177
ANKRD49	BC017798	−1.720	1.347	−1.610	2.315
CLPX	AL136922	−2.210	1.729	−1.614	2.400
FLJ20105	NM_001009954	−2.728	1.687	−1.942	2.462
CLK1	NM_001024646	−2.657	2.114	−1.858	2.785
FLJ20152	NM_001034850	−1.808	1.319	−1.906	3.105
CSTF2T	NM_015235	−2.424	1.505	−1.900	3.661
MCM10	NM_018518	−3.352	1.815	−2.109	3.887
ZNF550	BC034810	−2.594	1.597	−3.259	3.934

Comparison of the intersections of hyper- and microgravity-double-sensitive transcripts revealed 57 annotated transcripts differentially regulated in all conditions for the 19^th^ DLR parabolic flight and TEXUS-49 suborbital ballistic rocket campaigns. All transcripts were reversely regulated with respect to micro- and hypergravity conditions within the same platform. Positive sign: significantly up-regulated transcripts (p-value < 0.05, FC ≥ +1.3), negative sign: significantly down-regulated transcripts (p-value <0.05, FC ≤ −1.3). FCs are ratios between the averages of linear expression values. If the ratio is <1, FC is calculated as the negative reciprocal of the ratio.

### Almost complete adaptation of initially differentially altered transcripts

**Figure 10:**
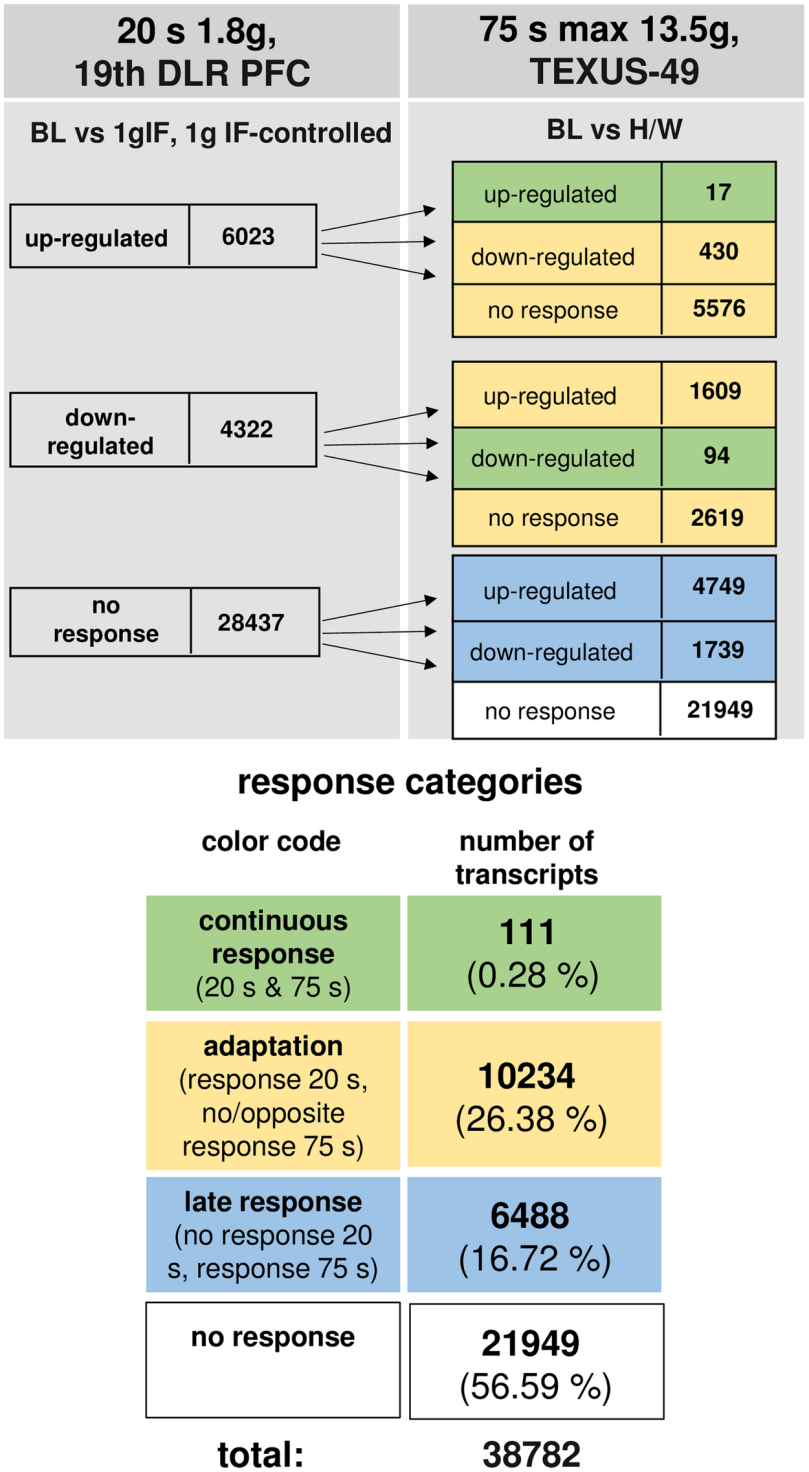
Time course of differential gene expression in hypergravity. Human myelomonocytic U937 cells were exposed to 20 s and 75 s of hypergravity. The numbers of annotated transcripts grouped according to their regulation after the two exposure times are shown. Continuous response means that annotated transcript are either up- or down-regulated at both time points. Adaption is either disappearance of the hypergravity induced effect or regulation in the opposite direction after 75 s. Late response is a regulation that only appears after 75 s. Most transcripts did not respond to hypergravity at both time points.

**Figure 11:**
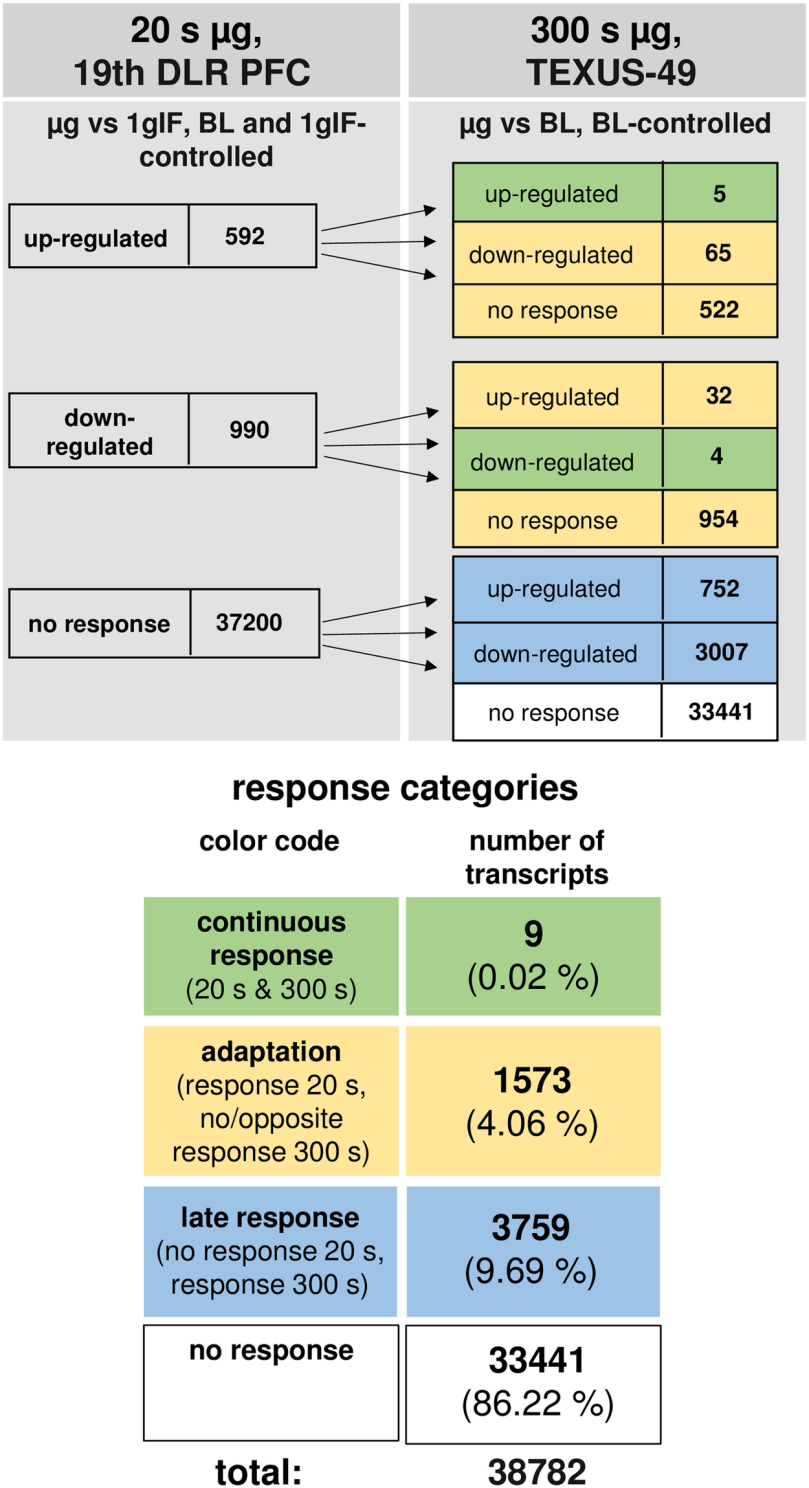
Time course of differential gene expression in microgravity. Human myelomonocytic U937 cells were exposed to 20 s and 300 s of microgravity. The numbers of annotated transcripts grouped according to their regulation after the two exposure times are shown. Continuous response means that annotated transcript are either up- or down-regulated at both time points. Adaption is either disappearance of the microgravity induced effect or regulation in the opposite direction after 300 s. Late response is a regulation that only appears after 300 s. Most transcripts did not respond to microgravity at both time points.

The time course of differentially regulated transcripts in hypergravity is summarized in Fig. 10 and in microgravity in Fig. 11. In hypergravity, a total number of 21949 transcripts (56.59%) were not altered at all. Out of 10345 initially altered transcripts after 20 s, only 111 transcripts (1.07%) were altered in the same direction after 75 s, whereas 10234 transcripts (98.92%) adapted. 6488 transcripts (16.72%) were altered after 75 s, but not after 20 s of hypergravity. In microgravity, a total number of 33441 transcripts (86.22%) were not altered at all. Out of 1582 initially altered transcripts after 20 s, only 9 transcripts (0.56%) were altered in the same direction after 300 s, whereas 1573 transcripts (99.43%) adapted. 3759 transcripts (9.69%) were altered after 300 s, but not after 20 s of microgravity. The nine non-adapting microgravity-sensitive transcripts were the transcription factor AKNA, histone-binding transcriptional repressor L3MBTL2, cytoskeletal protein TLN1, intracellular transport protein VPS29, receptor tyrosine kinase EPHA6 involved in RhoA regulation pathways, Fc receptor-like FCRLM1, regulator of phosphatidylcholine biosynthesis PCYT1B, and the RNA binding protein LOC643980 (Table 9). Thus, in our experiments, which have been conducted in two different experiment platforms (19^th^ DLR parabolic flight campaign and TEXUS-49 suborbital ballistic rocket campaign), we detected a significant high number of differentially regulated transcripts already after 20 s of microgravity or hypergravity. In microgravity, 99.43% of all initially altered transcripts adapted after 5 min. In hypergravity, 98.93% of all initially altered transcripts adapted after 75 s. Therefore, we identified two pools of altered transcripts: A first one, which responded after seconds and adapted within 5 min latest, and a second one, which appeared at least after 5 min of altered gravity.

**Table 9:** Differentially regulated annotated transcripts in microgravity identified in the 19^th^ DLR parabolic flight and TEXUS-49 suborbital ballistic rocket campaigns.

Gene name	Full gene name	Probeset ID	Gene function adopted from GeneCards HUMAN GENE DATABASE	Fold change 19^th^ DLR PFC µg vs BL-PFC hyp-g	Fold change TEXUS-49 µg vs BL-TX hyp-g
AKNA	AT-hook transcription factor	AB075848	RNA polymerase II core promoter proximal region sequence-specific DNA binding and transcriptional activator activity	1.627	1.670
LOC390988	hypothetical LOC390988	XM_372755	unknown function	1.655	1.524
L3MBTL2	l(3)mbt-like 2 (Drosophila)	AL136564	putative polycomb group (PcG) protein, maintains the transcriptionally repressive state of genes, involved in histone binding	1.414	1.610
TLN1	talin 1	BC020881	cytoskeletal protein that accumulates at cell-substratum and cell-cell contact sites, involved in actin filaments and assembly and cell spreading and migration, colocalisation with integrins in cell surface membranes enabling the attachment of adherent cells to extracellular matrices and of lymphocytes to other cells	1.330	1.806
VPS29	vacuolar protein sorting 29 (yeast)	BC032462	large multimeric retromer complex component, plays a role in retrograde transport of proteins from endosomes to the trans-Golgi network	1.334	1.332
EPHA6	EPH receptor A6	AY358738	receptor tyrosine kinase binding GPI-anchored ephrin-A family ligands on adjacent cells, important for G-protein signaling RhoA regulation pathway, and EPHA forward signaling	−1.404	−1.397
FCRLM1	Fc receptor-like and mucin-like 1	AF329495	receptor similar to receptors for the Fc fragment of gamma immunoglobulin	−1.336	−1.322
PCYT1B	phosphate cytidylyltransferase 1, choline, beta	NM_004845	regulation of phosphatidylcholine biosynthesis	−1.319	−1.511
LOC643980	similar to FRG1 protein (FSHD region gene 1 protein)	XM_928714	localizes to the nucleolus, involved in actin filament binding, poly(A) RNA binding, mRNA transport, and epigenetic regulation	−1.386	−1.757

Annotated transcripts with continuous response for up or down-regulation after 20 s and 300 s of microgravity are shown. Positive sign: significantly up-regulated transcripts (p-value <0.05, FC ≥+1.3), negative sign: significantly down-regulated transcripts (p-value < 0.05, FC ≤−1.3). FCs are ratios between the averages of linear expression values. If the ratio is <1, FC is calculated as the negative reciprocal of the ratio.

## Discussion

### Fast transcriptome response to altered gravity and potential gravity-sensitive cellular structures

One of the first theoretical studies about the physical background of cellular microgravity concluded that cells with a diameter of 10 μm and more would experience gravity^63^. However, because the weight of single normal-sized cells of 10 µm is too small compared with other cellular forces for direct “sensing” of the gravity vector^18^, gravity may thus be “sensed” indirectly at least as the magnitude of gravity-induced weight forces surrounding tissues and fluids^18^. Since decades it has been asked for mechanism by which the gravitational force becomes transmitted into a biological process^64^, but was rarely addressed in experimental approaches. In a very recent review about perception of gravity in eukaryotes^65^, gravity perception by cytoskeletal processes and mechanosensitive ion channels have been discussed, but the presented theories about gravitational force transmission in mammalian cells has not been validated experimentally so far. Therefore, we used a multi-platform approach to determine the time period between gravitational force change and transcriptome response, and also investigated the role of ion channels in gravitational force-induced transcriptome regulation. We were able to detect profound alterations in the transcriptome as early as 20 s after the onset of altered gravitational force. Therefore, the preceding hypothetical transduction processes have to be fast enough for inducing transcriptome changes within 20 s of force alteration. We therefore compiled the transduction velocities of potential transduction processes: DNA decondensation was shown to occur within the time frame of minutes to hours^66^, transcription factor binding takes a couple of seconds^67^, and the elongation rate of RNA polymerase II was found to range between 0.37 kb/min^68^ and 4.3 kb/min^69^ with a median rate of 2.1 kb/min^68^. Theoretical velocities even above 50 kb/min have been calculated^70^. Therefore, significant transcription-caused transcriptome alterations are theoretically possible after 20 s, if (1.) the particular chromatin regions are already decondensed and (2.) the preceding signal cascade requires only seconds. Such high transduction velocities have already been demonstrated for direct mechanotransduction processes into the nucleus^17,71,72^.

### Biochemical mechanotransduction pathways

Biochemical mechanotransduction can occur through sensing of the major matrix signals via focal adhesion proteins and the cytoskeleton^73–75^, leading to the activation of a variety of transcription factors such as YAP/TAZ^76–79^, SRF/MRTF-A^80,81^, NF-kappaB^82^, and JMY^83^ and transcription co-activators^84,85^ to regulate gene expression. Interestingly, SRF binding sites are present in the promoters of the majority of genes inhibited in T cells activated in microgravity^24^. In summary, mechanosensation at the plasma membrane leads to downstream nucleocytoplasmic shuttling of various transcription regulators^17^. Thus, force transduction along activation of transcription factors is very well described and a potential reason for fast transcriptome response to altered gravity, but requires 10 min or more into the nucleus^86,87^, probably not fast enough to explain rapid transcriptome alterations after 20 s. In this context, experiments with the TRPC channel inhibitor SKF-96365 revealed that only a very low share of all detected gravity-induced transcriptome alterations can be attributed to this type of ion channels.

### Transcriptional or posttranscriptional regulation processes after rapid mechanical transduction

Fast transcriptome alterations could be initiated also at the posttranscriptional level: RNA binding proteins (RBPs) bind mature mRNA and exert important regulatory effects on mRNA stability and translation. RBPs can either function to promote mRNA degradation as in the case of AUF1 or TTP shortening the mRNA half-life, or they promote mRNA stability like HuR extending the mRNA half-life. Furthermore, RBPs have been shown to work cooperatively with miRNAs to regulate mRNA turnover. For example, HuR is able to recruit let-7/RISC to inhibit c-MYC mRNA translation^88^ and it competes with miR-494 and miR-548c-3p for the binding of nucleolin and TOP2A mRNA, respectively^89,90^. RBPs were found to be associated to the cytoskeleton^91,92^ and play critical roles in the localization of mRNA^93^. Apart from that, it has been observed that binding of the extracellular matrix to integrins on the cell surface and mechanical tension induce movement of mRNA to focal adhesions^94^. These findings suggest that mechanical stimuli act on RBP function and thus on mRNA regulation. mRNAs that are translationally repressed, have been found to accumulate in cellular structures called P- or GW-bodies where they are degraded^95,96^. Alternatively, mRNA can be stored as a response to stress conditions in cytoplasmic aggregates named stress granules (SGs)^97^. In P-bodies proteins are enriched that play an important role in RNA deadenylation, decapping and degradation. Interestingly, we identified the 5′-3′ exoribonuclease xrn1, representing a key player in the cellular mRNA degradation process, being differentially regulated when exposed to altered gravity. Furthermore, several other genes involved in RNA splicing, transcriptional regulation and RNA processing were found highly sensitive to gravitational changes. miRNAs, which were recently found differentially expressed after 20 s of microgravity in Jurkat T cells^29^, appear to repress translation and promote decay by recruiting P-body components to individual mRNAs^95^. SGs and P-bodies share protein components and can be found in close vicinity in the cell, possibly interchanging their load^98,99^. According to the hypothetical model of Wilczynska and colleagues, cells react upon stress with a translational block or translational repression including the storage of mRNAs in stress granules. The SGs can either revert when the cell adapted to the situation or reached normal conditions or the mRNAs can be degraded^99^. The cellular pause of protein synthesis triggered by environmental stress conditions (heat, UV irradiation, osmotic pressure etc.) is important for the cell to focus on repair processes, e.g. due to DNA damage. The translational stress that follows the environmental stress is highly specific for different types of genes. While some selected groups of RNAs are stabilized, others are destabilized and degraded^100^. In the hypothetical model presented by Anderson and Kedersha^97,101^ it is shown that the sorting of mRNAs into SGs or P-bodies is reversible, meaning that the remaining RNAs can return to the polysome fraction after the cell reaches the equilibrium again. This process of re-distribution occurs rapidly and shuttling times in and out of SGs have been measured with a half-life between 2 and 8 seconds^98^. Therefore, significant SG-bodies-caused transcriptome alterations are theoretically possible after 20 s, if the preceding signal cascade to the cytoplasm requires only seconds.

In conclusion, while biochemical force-transduction processes appear unlikely to explain the rapid transcriptome response, all hypothetical mechanisms such as transcriptional regulation, RBPs or P-body or SG-based regulations are only fast enough if followed by rapid mechanical transduction either into the nucleus or into the cytoplasm lasting in the range of microseconds^71^. To explain how cells can not only sense but also spatially transfer physical forces over relevant distances, the model of “tensegrity” architecture of the cytoskeleton implies that cytoskeletal components are interconnected with a certain level of tensional pre-stress, established by a force balance between extracellular adhesions, contractile microfilaments and microtubules^6,8,102^. This architecture not only provides shape stability to the cell, but it also implicates that tensional forces are propagated across the whole tensegrity structure which includes intracellular structures^102–104^. Propagation of stress along cytoskeletal filaments takes only 2 µs to cover 50 µm, whereas diffusion-based biochemical signaling through the cytosol needs seconds to span the same distance^71^.

### Rapid force transduction chains to the chromatin via the cytoskeletal-nucleoskeletal network

Although the majority of mechanotransduction research has focused on the perception of mechanical forces at and across the cell membrane to induce signaling pathways originating in the cytoplasm^17,71^, many experimental evidences demonstrated a very close connection between cytoskeletal force transduction and chromosome organization and gene expression: Alterations in cell geometry resulted in cytoskeletal reorganization, leading to nuclear morphology remodeling, affecting orientation, 3D radial position, compaction, and intermingling of chromosome territories^16^ and chromatin condensation^105^, accompanied by differential gene expression patterns^106^. Force transmission through the actin cytoskeleton to the nuclear envelope resulted in direct displacements of Cajal body-associated protein complexes^107^ and through the LINC complex to chromatin deformation and force-induced expression up-regulation of specific genes^14^. Therefore, mechanical forces can regulate gene expression independent of molecular relays by opening or closing chromatin configurations^72^. Mechanical force transduction into the chromatin seems to occur within seconds, subsequently resulting in transcription alteration proportional to the magnitude of chromatin stretching^12^ and was observed to induce epigenetic changes in promoter regulation^108^. Nuclear lamins, emerin, LINC complex proteins, heterochromatin protein-1 (HP1) and BAF have been recently proposed to be involved in force transduction to the chromatin, resulting in stretching and increased accessibility of the transcriptional machinery^12^. BAF binds directly to linker histone H1.1 and core histone H3 *in vitro* and *in vivo*^109^ and is associated with poly(ADP-ribose) polymerase-1 (PARP-1)^110^, a central modulator of chromatin structure and transcription^111^. Interestingly, PARP-1 has been demonstrated to be involved in hyperstretch-induced mechanotransduction in bronchial epithelial cells^112^. PARP-1 and PARP-2 also interact physically and functionally with HP1^113^. Importantly, HP1 binds to histone H3 methyl lysine 9 and SuvH39, a histone H3 lysine methyltransferase, where methylation of histone H3 lysine 9 by SUV39H1 creates a binding site for HP1 proteins^114^ bridging H3K9me3 in condensed chromatin^115^. Because silencing H3K9 methyltransferase SUV39H1 completely blocked the force-induced H3K9 methylation^116^, and H3K9me3 has been shown to be mechanical strain-driven^13^, Suv39H and H3K9me3 are potential components of the gravity-induced force transmission cascade to the chromatin.

Surprisingly, more than 98% of all initially altered transcripts adapted rapidly to the new gravitational environment after 75 s of hypergravity or 300 s of microgravity, respectively. Thus, the same gravity condition obviously resulted in qualitatively different directions of transcript regulation as a function of time. Gene ontology enrichment analyses (Supplementary Figs 1–4) revealed regulatory effects of altered gravity on RNA binding, regulation of transcription and posttranscriptional regulation of gene expression. In this context, the transcription factor AKNA, the histone-binding transcriptional repressor L3MBTL2, and the RNA binding protein LOC643980 belong to the group of non-adapting microgravity-sensitive transcripts (Supplementary Table S3). Although molecular mechanisms of rapid transcriptome adaptations to altered gravity are unknown, they appear probably at different steps of transcriptional processing. Since the macrophageal oxidative burst reaction adapted rapidly to microgravity in only 42 s^39^, we assume rapid adaptation mechanisms are occurring not only inside the nucleus, but also in the cell membrane. It could be therefore possible, that the cytoskeletal-nucleoskeletal network is reacting to gravitational force alterations as a whole interconnected system.

### No pro-apoptotic/necroptotic effects in altered gravity

In order to exclude potential pro-apoptotic/necroptotic effects during the entire cell preparation process for the experiment mission and to investigate potential pro-apoptotic effects of altered gravity as described earlier^60,61^, we analyzed apoptotic/necroptotic genes identified from the KEGG database and from relevant literature and revealed only slight transcript expression differences for them. We compared these values to previously published ones of apoptotic gene expression in human monocytes displaying fold changes of >+5 for caspase 6^117^, in human chronic myelogenous leukemia K562 cells with fold changes of +1.779 and +1.633 for caspase 6 and caspase 8 respectively after chemical stimulation^118^, and various apoptosis induced genes in U937 cells showing fold changes >+ 2 after a combination of thermal and chemical stress^119^. In comparison to this data we observed a rather mild cellular reaction in altered gravity (Supplementary Tables S3 and S5). Additionally, all microgravity-altered pro-apoptotic/necroptotic transcripts adapted after 5 min, as well as all hypergravity-altered transcripts, except of FADD (BC000334). However, FADD is down-regulated after 20 s and 75 s of hypergravity, indicating a rather protective effect of the altered gravity condition. In this context, transient up-regulation of apoptotic markers with a subsequent reversibility and cellular recovery has been described previously^120–122^.

The experiments of this study were performed in different very low gravity environments (10^−2^–10^−3^ g for parabolic flight experiments and 10^−4^ g for TEXUS experiments). Currently, it is unknown, in which extent different levels of very low gravity are transduced into a cellular response. Whereas a 2D clinostat study with 1F6 melanoma cells reported differences in guanylyl cyclase A mRNA expression in the range between 0.012–0.036 g^123^, the response of the oxidative burst reaction in NR8383 macrophages did not differ between the range of 10^−2^–10^−3^ g for parabolic flight experiments 35 and the <10^−5^ g for the ISS experiment^39^. Additionally, indications for a gravitational threshold between 0.3 g and 0.5 g were found^39^. Therefore, all microgravity levels are probably below this threshold, but current knowledge about biological effects of gravitational changes in very low gravity environments is limited. A recent study re-analyzed transcriptome data from two space experiments (ISS, human T cells and Progress 40 P, HUVEC) and two ground-based studies with “simulated” microgravity (humans T cells and PBMC) and identified the reduction of second messenger molecules generation as a statistically significant pathway in all datasets, which has been discussed as potential effect of microgravity-induced low shear stress^124^. Due to the rapid response and adaptation of the transcriptome (Figs 10 and 11), the described alterations after longer microgravity exposure time could re-represent a stable systemic regulatory status, a steady-state, and therefore the last and final phase of microgravity-induced transcriptome alterations. Of course, the rapid cellular response to altered gravity^35,39^, detected in cells of the monocyte-macrophage system in this study, might not be present in other cell types, particularly if caused by cell-type specific chromosome territory architectures^16,105,106^.

In conclusion, we detected an immediate transcriptome response after 20 seconds of altered gravity, which in turn adapted rapidly afterwards. Theoretical explanations for rapid transcriptome regulation require rapid transduction into the nucleus, which is likewise theoretically possible and has been demonstrated for other mechanical forces than gravity. Thus, we assume that gravitational forces are rapidly and constantly transduced into the nucleus, acting as an omnipresent condition in the nuclear and chromatin structure and subsequently resulting in homeostasis of gene expression. Due to the proposed fundamental role of gravitational forces for transcriptome homeostasis, the existence of rapid adaptation mechanisms is not surprising. Gravity could potentially represent an essential environmental condition for the genomic functionality of life on Earth.

## Material and Methods

### Cell culture

The human myelomonocytic cell line U937 (ATCC CRL1593.2) was used as a model cell line to analyze the differential gene expression under altered gravity conditions in the human monocyte-macrophage system. U937 cells were cultured in RPMI-1640 (Biochrom/Merck Millipore, Germany), supplemented with 10% fetal bovine serum (FBS Superior; Biochrom/Merck Millipore, Germany), 1% glutamine (200 mM; Gibco/Life Technologies, Germany) and 1% penicillin/streptomycin (10,000 U/ml and 10,000 µg/ml respectively; Gibco/Life Technologies, Germany). Cells were cultured with a density of 0.2 × 10^6^ cells/ml and medium exchange was performed every 48 hours. Cells were centrifuged at 300 g for 5 min at room temperature, the supernatant was discarded, and the cell pellet was resuspended in fresh medium. An aliquot was taken, diluted with trypan blue solution and the number of vital cells was counted. Cells were reseeded at a concentration of 0.2 × 10^6^ cells/ml in fresh medium.

### Parabolic flight experiment platform

As described previously^29^, parabolic flights are an ideal platform to study initial and primary effects in mammalian cells and the associated rapid responsive molecular alterations excluding influences and interferences of secondary signal cascades. Parabolic flights offer a sequence of consecutive gravity conditions including 1 g, 1.8 g, and microgravity (µg) with a quality of 10^−2^ to 10^−3^ g. We designed and constructed an experimental system which allows cell culture experiments during parabolic flights on board the Airbus A300 ZERO-G (reg. no. F-BUAD), that has been used already for different parabolic flight experiments^28,29,36^. Primary importance was placed on realizing the direct safety technique during the development activity. The experimental structure (Fig. 1c) consists of three experiment racks (storage rack for cell culture containers before the experiments at 36.5 °C, cooling rack for storage of cell culture containers after cell lysis at 4 °C, and a working rack for handling and execution of the experiments). The modular system is able to accommodate up to 54 cell culture containers (double containment) for each flight and allows storage of cell cultures until the start of the experiment, injection of a fluid (culture medium) at any defined time during the parabolic maneuver, and automatic injection of a second fluid (lysis buffer) after 20 s at the end of a defined gravity phase. Appropriate in-flight controls were obtained during the 1 g flight phase directly before the first parabola. Injection of all fluids operates automatically and is pre-programmed, while exchange of cell culture containers and supervision of the experiment was performed manually. During the 19^th^ DLR parabolic flight campaign (PFC), we investigated the gene expression in human U937 cells in microgravity and hypergravity (1.8 g) compared to in-flight 1 g. Experiments were only conducted during the first parabola to assure that detected differential gene expression levels were a result of the effect of gravitational change and not an accumulated long-term effect.

### Preparation and execution of the parabolic flight experiments

During the 19^th^ DLR PFC, 1 × 10^7^ U937 cells in 10 ml medium (RPMI 1640 supplemented with 1% penicillin/streptomycin, amphotericin (Gibco/Life Technologies, Germany), 1% glutamine and 2% FBS (i.e. serum starved) were filled into 200 ml Nutrimix bags (B. Braun Melsungen, Germany) and transported from the home laboratory to the pre-flight preparation laboratories at the NOVESPACE premises in Bordeaux, France. After arrival, cells were de-starved with 0.8 ml FBS per sample and stored at 36.5 °C overnight and used for the flight experiment on the following morning. 36.5 °C were chosen instead of 37 °C to rule out any thermic activation of the cells caused by regulatory oscillation of the storage rack. For the flight day, the Nutrimix bags were placed in a solid plastic housing to create a double containment that prevents spillage of fluids in the aircraft in case of leakage of the hardware system. Rapid lysis of U937 cells in the respective gravity phase was achieved by fast injection of 5 volumes of RLT buffer (Qiagen, Germany) and mixing by inverting the samples three times immediately at the appropriate time point (1 g in-flight samples 5 min before the first parabola, 1.8 g and microgravity samples during the first parabola). After landing, 1 g ground controls were performed immediately using the same hardware inside the aircraft. Post-flight, all samples were directly transported to the on-site laboratory where total RNA was purified. In total, 28 samples were obtained from two parabolic flight days: 6x H/W 1 g GC, 8x 1 g IF, 6x BL-PFC hyp-g, 8x µg (see Table 1).

### RNA isolation after the parabolic flight

RNA was isolated as described previously^29^. After landing of the aircraft and transport of the samples to the laboratory facilities, the protective plastic housings were disassembled, the Nutrimix bags were gently agitated and the lysed cell solution was filled into a T75 straight neck cell culture flask. The cell solution was mixed for 10 s by vortexing and sheared by passing four times through a Ø 0.8 × 120 mm needle (B. Braun Melsungen, Germany) fitted to a sterile 50 ml syringe. 50 ml of absolute ethanol were added and precipitates were resuspended by vigorous shaking. A Qiavac 24 plus vacuum system (Qiagen, Germany) was prepared by placing 24 valves and sterile connective pieces on the Qiavac 24 plus vacuum manifold and an RNA maxi column (Qiagen, Germany) was attached to each connective piece. The system was set to a vacuum level of -200 mbar and the RNA maxi columns were loaded with the lysed cell suspensions. Subsequently, the valves were closed and the RNA maxi columns were centrifuged at 3220 g for 3 min at room temperature. The, 15 ml of buffer RW1 (Qiagen, Germany) were carefully applied to the column to wash the membrane-bound RNA. After centrifugation at 3220 g for 7 min at room temperature, the flow through was discarded and additional two washing steps were performed with 10 ml RPE buffer (Qiagen, Germany) followed by centrifugation at 3220 g for 3 min and 10 min at room temperature, respectively. The column-bound RNA was eluted by application of 600 µl of pre-warmed RNase-free water (Qiagen, Germany), incubation for 1 min at room temperature and centrifugation for 4 min at 3220 g again at room temperature. The elution step was repeated with the first eluate, the column was centrifuged for 7 min at 3220 g, and the purified RNA was stored in a sterile 1 ml cryotube on dry ice. Finally, the extracted RNA was transported on dry ice and stored at −80 °C until the processing of the RNA for the microarray analysis.

### TEXUS-49 suborbital ballistic rocket experiment

TEXUS suborbital ballistic rockets consist of a two-stage VSB-30 rocket (S-30 solid rocket-stage engine with S-31 second-stage engine) and the payload (weight 390.4 kg, length 5083 mm). TEXUS-49 was launched on March 29^th^, 2011 at 06:01 a.m. from the ESRANGE (European Space and Sounding Rocket Range) Space Center near Kiruna, Sweden, north of the Arctic Circle. During the ballistic suborbital flight, an altitude of 268 km and 378 s of microgravity with a quality of better than 10^-5^g were achieved. Further parameters include: First stage peak thrust acceleration of 6.3 g, mean thrust acceleration of 5.03 g, first stage burnout at 12.3 s, engine separation at 13.6 s, second stage peak thrust acceleration of 13.5 g, mean thrust acceleration 7.30 g, burnout at 43.0 s, yo-yo despin at 56.0 s, engine separation at 59.0 s. At ESRANGE, fully equipped laboratories enabled complete on-site preparation of the biological experiments, integration of the experiment into the payload platform 1 h before launch, and autonomous experiment execution in a programmed sequence. At the end of the free-fall period, the payload reentered the atmosphere and returned to the ground after parachute deployment at 5 km altitude and with a sink velocity of 8 m/s. The experimental unit was immediately recovered and returned to the launch site within 1.5 h after lift-off by helicopter. The general experimental composition consists of multiple sets of three syringes, filled with cell suspension (human U937 cells), cell culture medium with or without SKF-96365, and lysis solution (Trizol LS). All three syringes were connected by a T-piece, while small plugs at the outlet ports prevented premature contact of the fluids. The syringe systems were housed in a temperature-controlled, vacuum-resistant container (Fig. 1d). The temperature-controlled syringe systems were placed at microgravity positions inside the payload structure. Before launch and during flight, syringes were activated by a pneumatic system at pre-set time points. Several pre-flight tests and development tests were conducted: Biocompatibility tests, chemical stability tests, culture medium optimization with regard to buffer systems and supplements, sterilization tests, viability tests, cell lysis tests (different lysis compounds and concentrations). The entire mission procedure was standardized and tested several times. Margins and possible holding times were determined. The experimental setup consisted of the baseline group (lysis after hypergravity phase and before onset of microgravity), in-flight microgravity group (lysis after 5 min of microgravity and before reentry into the Earth’s atmosphere), and 1 g ground control reference inside the experimental hardware. Cells, medium and lysis fluid (Trizol LS) syringes were prepared directly before the launch. All procedures started 7 hours before launch. The experimental containers were integrated into the payload structure by a “late access” port between 1:15 h and 0:45 h before launch. Sample temperature was maintained at 36.5 °C ± 0.5 °C until lysis. On landing and payload recovery, the experimental containers were immediately removed and returned to the ESRANGE laboratory for further processing. The cell suspension was transferred from the syringes into sterile plastic reaction tubes, and cells were homogenized with subsequent isolation of RNA. The purified RNA was stored and transported on dry ice or in liquid nitrogen and analyzed afterwards by means of genome-wide expression arrays.

### Experimental preparation and integration for TEXUS-49

U937 cells were cultured in the ESRANGE laboratories on site. Cells were cultivated with a density of 0.2 × 10^6^ cells/ml, and the medium was exchanged every 48 hours (see above). During the countdown phase, cells were visually inspected, harvested, the vital cell number was counted, and cells were pooled to a concentration of 5 × 10^7^ cells/ml. 0.5 ml of cells (i.e. 25 million cells) were filled in sterile 3 ml plastic syringes shortly before the handover to the launch team. Additionally, a second set of syringes was filled with 0.3 ml of cell culture medium with or without 0.3 ml SKF-96365 (25 µM; Sigma Aldrich, Germany), and a third set with 1 ml Trizol LS (Life Technologies, Germany) per sample unit. The three syringes with small plugs at the outlet ports were mounted on a sterilized plastic T-block with a connecting tubing system. 26 experiment units were prepared and stored at 36.5 °C ± 0.5 °C until integration into the payload of the rocket or until manual execution of the ground controls, respectively. These experimental units were finally integrated into the automatically operated experiment system. During the experimental run, firstly 0.3 ml of cell culture medium with or without SKF96365 and secondly 1 ml of Trizol LS were injected to the cell suspension at defined time points to lyse the cells and preserve the current status of differential gene expression. Directly before the µg phase, a set of samples was lysed at the time point of 75 s after launch (baseline, BL), representing the effect of hypergravity, spin and vibrations during the launch and rocket engine burn. Two further sets of samples (with and without SKF-96365) were fixed at 375 s after launch, shortly before the end of the µg phase. Additionally, 1 g ground controls were kept on ground in the incubator analogously to the µg sample group. In total, 26 samples were obtained after the TEXUS-49 rocket flight: 6x H/W 1 g GC, 5x BL-TX hyp-g, 7x µg, 4x µg SKF, 4x H/W 1 g GC SKF (see Table 1).

### RNA isolation after TEXUS-49 landing

The sample processing has been described previously^29^. Directly after landing, localization and recovery of the payload by helicopter, the experiment modules were dismantled and handed over for processing. The sample containing syringes were connected to a sterile 20G needle (B. Braun Melsungen, Germany), the 1.8 ml of cell suspension were sheared three times and distributed equally in two 2.0 ml reaction tubes. 0.1 ml of chloroform (Sigma-Aldrich, Germany) were added, the homogenate was vortexed for 15 s and incubated for 5 min at room temperature before a 15 min centrifugation step at 11000 g and 4 °C. The upper phase from both 2.0 ml tubes was transferred into one 15 ml tube and 4 ml of RLT buffer (Qiagen, Germany), as well as 3 ml of absolute ethanol were added and the suspension was mixed. 4 ml of this solution were pipetted on an RNA Midi column (Qiagen, Germany) and centrifuged for 30 s at 3000 g and room temperature. The flow through was discarded and the residual 4 ml of RNA solution were loaded on the column. All samples were centrifuged for 5 min at 3000 g at room temperature. Then, the columns were washed twice with 2.5 ml of RPE buffer (Qiagen, Germany) and centrifuged firstly for 2 min and additionally for 5 min at 3000 g at room temperature. The RNA was eluted by the addition of pre-warmed 250 µl RNase-free water (Qiagen, Germany) to the column, incubation for 1 min at room temperature, and centrifugation for 3 min at 3000 g and room temperature. The flow through was loaded again onto the column, incubated for 1 min at room temperature, and centrifuged for 5 min at 3000 g and room temperature. The isolated RNA was transferred into sterile 1 ml cryotubes and stored and transported at −80 °C. After arrival in the home laboratory, samples were stored at −80 °C until the processing of the RNA for the microarray analysis.

### RNA sample processing and microarray data analysis

The RNA quantity and quality of the samples of the 19^th^ DLR parabolic flight campaign and the TEXUS-49 sounding rocket mission was analyzed using a Nanodrop 1000 (Thermo Scientific). All RNA samples were of high quality with 260/280 nm ratios between 1.9 and 2.1. The RNA integrity number (RIN) was measured using an Agilent 2100 Bioanalyzer (Agilent Technologies, USA) and only RNA samples with a RIN of >8.7 were used for the following microarray analysis. 400 ng total RNA were cy3-labeled with the Low RNA Input Linear Amplification Kit, PLUS, One-Color“ (Agilent Technologies) and hybridized for 17.5 h to a NimbleGen expression microarray (12 × 135,000 features) applying the “Gene Expression Hybridization Kit” (Agilent Technologies, USA). Microarrays were washed and scanned with the Micro Array Scanner G2505B (Agilent Technologies, USA). The image files of the scanner were analyzed with the NimbleScan Software 2.6 using the Robust Multi-Array Analysis (RMA) with default parameters. RMA represents a probe-level summarization method that identifies probes that are outliers in the overall behavior of the expression measured for a given gene. Differential expression of transcripts was determined based on the normalized microarray data using Excel 2013 and expression fold changes (FCs) of all transcripts on the microarray were calculated. FCs were used for comparisons of experimental groups within one experimental platform. For this, averages of the linear expression values were determined for each experimental group. The ratio was calculated by dividing the average value of one experimental group by the average value of the experimental group to which it should be compared. If the ratio is ≥1 it is equal to the FC, if the ratio is <1 the FC was determined by building the negative reciprocal of the ratio. Furthermore, t-tests were performed for all comparisons. FCs ≤ −1.3 or ≥1.3 with p-value < 0.05 were regarded to represent a significantly differential expression.

### Intra-platform and inter-platform comparisons

To further validate the relation between the different gravitational conditions and the detected differential expressions, intersections were made between the experimental comparison and respective control-comparison whenever available. Transcripts which were differentially expressed in the experimental and in the control-comparison were consequently excluded from the pools of gravisensitive transcripts (Tables 3, 5, 7, 8 and Venn diagrams). To analyze if transcripts respond preferentially to hyper- or to microgravity or to both conditions, intersections were made between the pools of hypergravity- and microgravity-sensitive transcripts. Furthermore, to evaluate the gravisensitivity of transcripts over time, intersections were made between the pools of gravisensitive transcripts of both experimental platforms.

### Gene annotation enrichment analysis (GOrilla analysis)

The gene annotation enrichment analysis was carried out by using DAVID 6.8. Enriched GO terms visualized in ranked lists of genes were generated using GOrilla^125^. For illustration purposes only end points of processes and functions are summarized. The p-value threshold was set to <0.05.

## Electronic supplementary material

GOrilla analysis

## Data Availability

The datasets generated and analyzed during the current study can be accessed in the GEO (Gene Expression Omnibus) repository (www.ncbi.nlm.nih.gov/projects/geo) under accession no. GSE101309.
