# Supplementary material for: Rapid coupling between gravitational forces and the transcriptome in human myelomonocytic U937 cells
Source: Sci Rep. 2018 Sep 5;8:13267. doi: 10.1038/s41598-018-31596-y (PMC6125427; doi:10.1038/s41598-018-31596-y)
Supplement: Supplementary file 1 — GOrilla analysis [file 41598_2018_31596_MOESM1_ESM.pdf]

Supplementary Information

Rapid coupling between gravitational forces and the transcriptome in human myelomonocytic U937 cells

Cora S. Thiel [a,b # *], Svantje Tauber [a,b #], Swantje Christoffel [a,b], Andreas Huge [c], Beatrice A. Lauber [a], Jennifer Polzer [a], Katrin Paulsen [a], Hartwin Lier [d], Frank Engelmann [d,e], Burkhard Schmitz [f], Andreas Schütte [f], Christiane Raig [a], Liliana E. Layer [a] , Oliver Ullrich [a,b,g *]

[a] Institute of Anatomy, Faculty of Medicine, University of Zurich, Winterthurerstrasse 190, 8057 Zurich, Switzerland

[b] Department of Machine Design, Engineering Design and Product Development, Institute of Mechanical Engineering, Otto-von-Guericke-University Magdeburg, Universitätsplatz 2 , 39106 Magdeburg, Germany

[c] Core Facility Genomic, Medical Faculty of Muenster, University of Muenster, Albert-Schweitzer-Campus 1, D3, Domagstrasse 3, 48149 Muenster, Germany

[d] KEK GmbH, Kemberger Str. 5, 06905 Bad Schmiedeberg, Germany

[e] Ernst-Abbe-Hochschule Jena, Carl-Zeiss-Promenade 2, 07745 Jena, Germany

[f] Airbus DS GmbH, Airbus-Allee 1, 28199 Bremen, Germany

[g] Zurich Center for Integrative Human Physiology (ZIHP), University of Zurich, Winterthurerstrasse 190, 8057 Zurich, Switzerland

Supplementary Fig. S1: GOrilla analysis of microgravity-sensitive differentially expressed transcripts identified for the 19th DLR parabolic flight campaign.

Supplementary Fig. S2: GOrilla analysis of hypergravity-sensitive differentially expressed transcripts identified for the 19th DLR parabolic flight campaign.

Supplementary Fig. S3: GOrilla analysis of microgravity-sensitive differentially expressed transcripts identified for the TEXUS-49 suborbital ballistic rocket campaign.

Supplementary Fig. S4: GOrilla analysis of hypergravity-sensitive differentially expressed transcripts identified for the TEXUS-49 suborbital ballistic rocket campaign.

Parabolic flight experiment

microgravity-sensitive differentially expressed transcripts

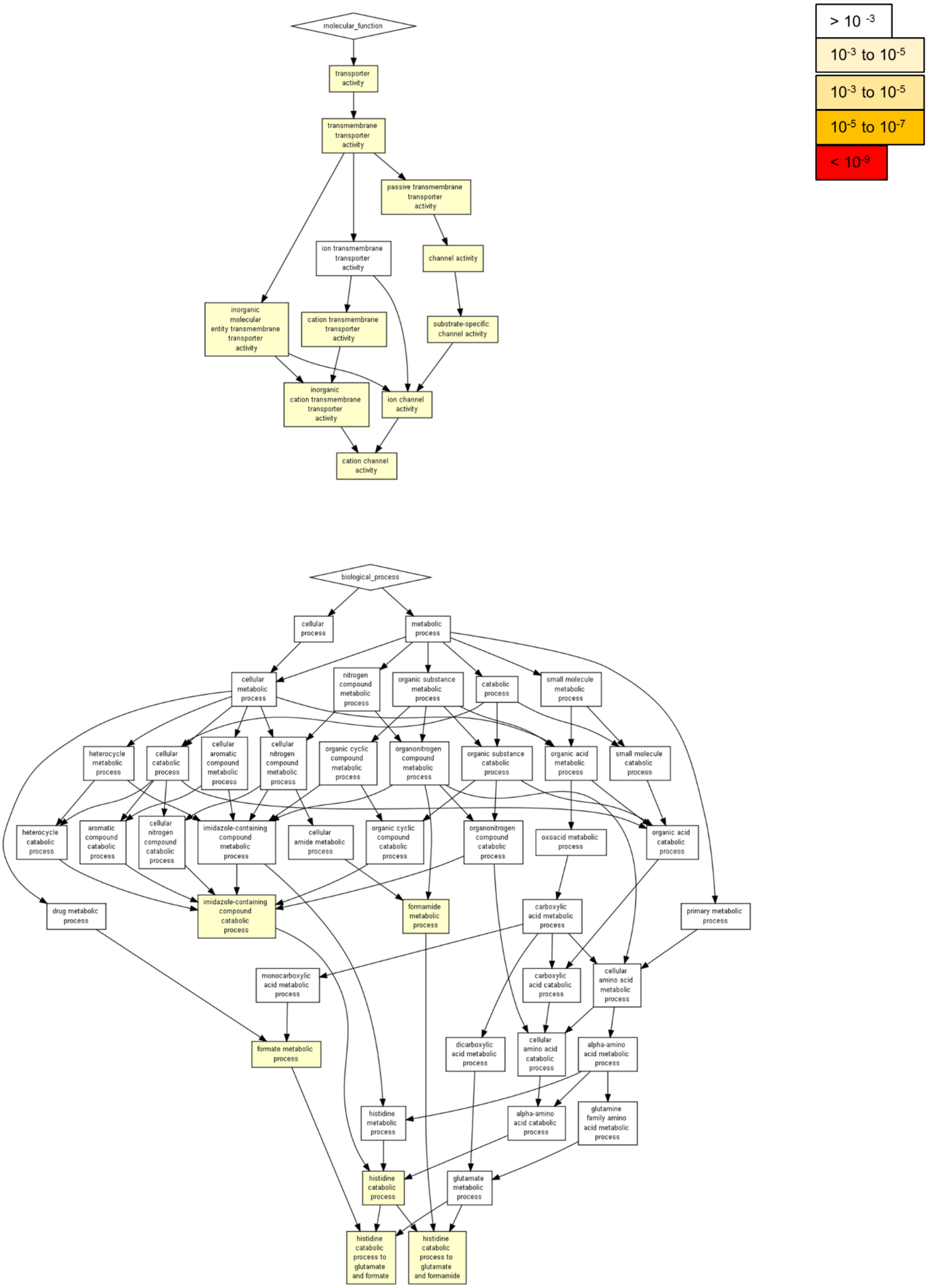

Supplementary Fig. 1

Parabolic flight experiment

hypergravity-sensitive differentially expressed transcripts

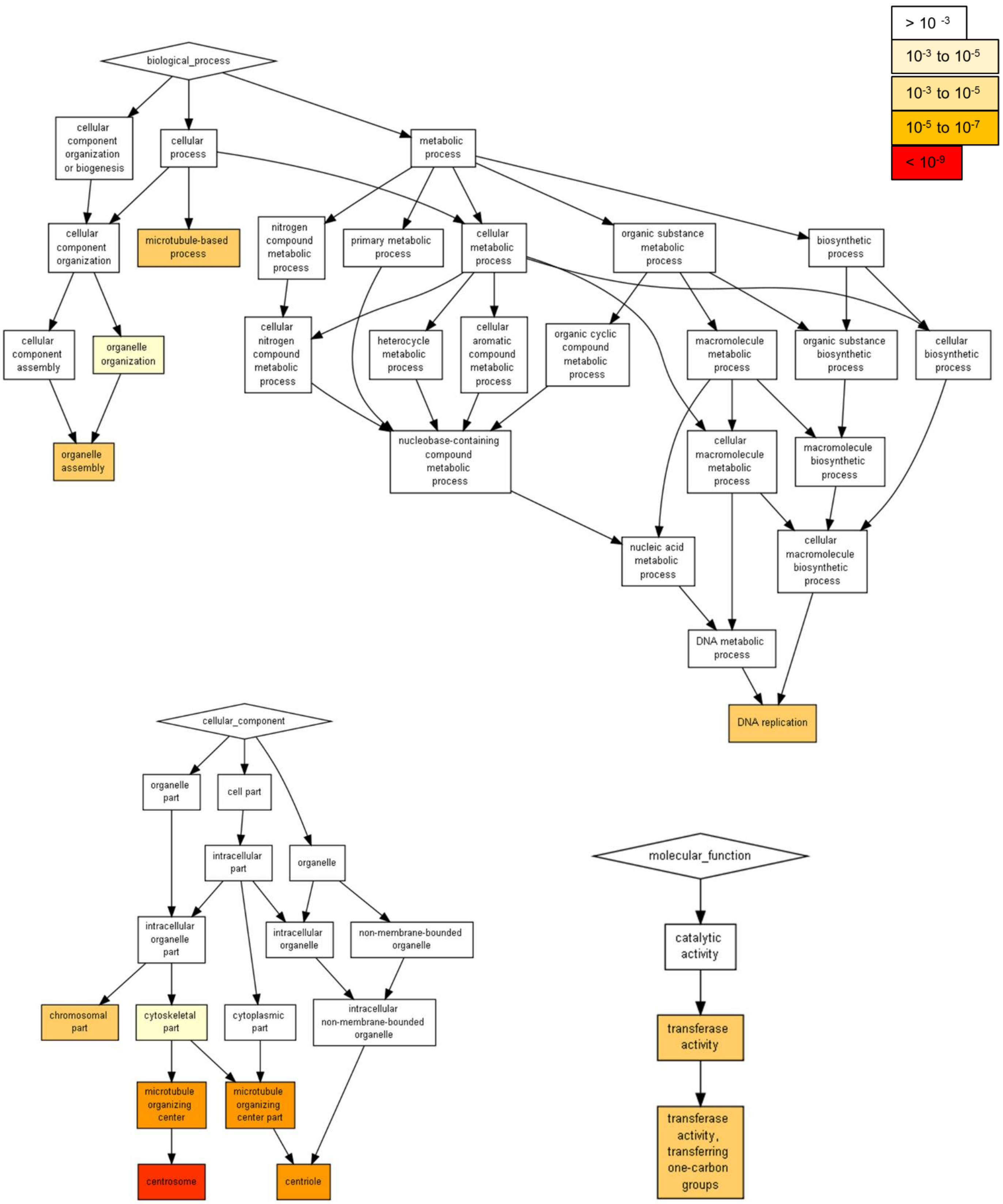

Supplementary Fig. 2

TEXUS-49 suborbital ballistic rocket experiment

microgravity-sensitive differentially expressed transcripts

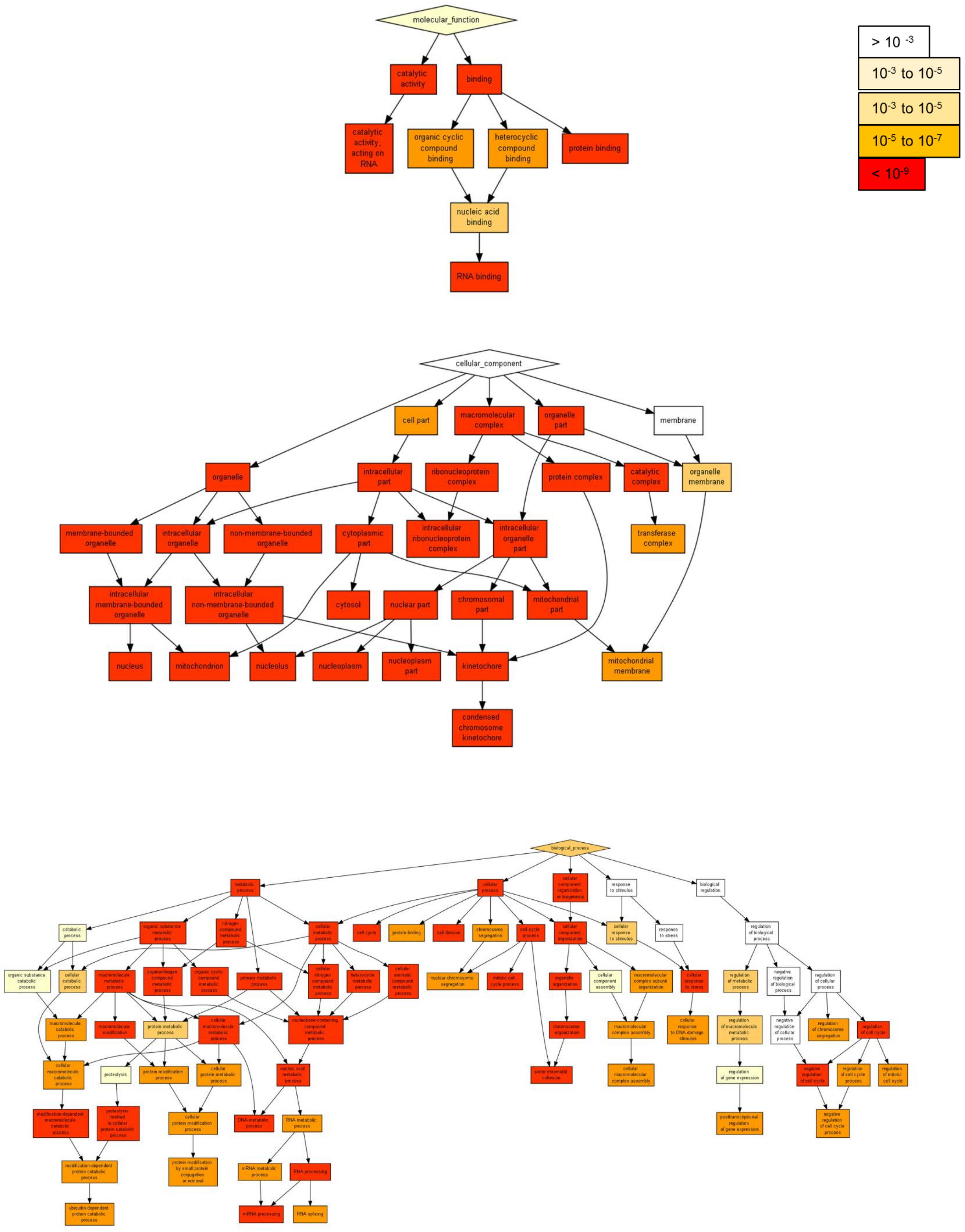

Supplementary Fig.3

TEXUS-49 suborbital ballistic rocket experiment

hypergravity-sensitive differentially expressed transcripts

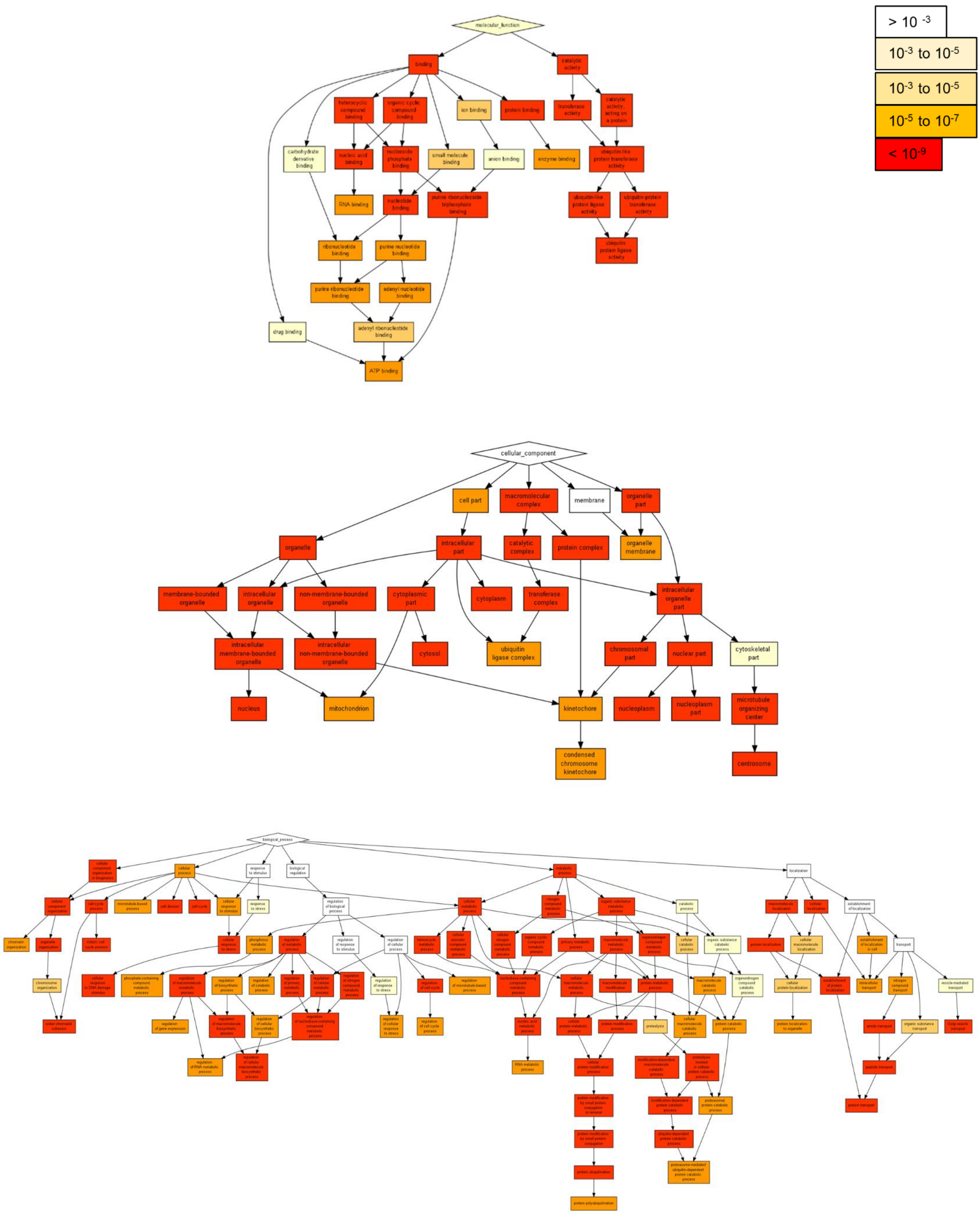

Supplementary Fig.4

Supplementary Table S1: Differentially regulated annotated transcripts in microgravity (alphabetically listed), sensitive to the cation channel inhibitor SKF-96365, identified in the TEXUS-49 suborbital ballistic rocket campaign. Annotated transcripts with a sensitivity to microgravity which is either enhanced or attenuated by SKF-96365 exposure are shown. Green: significantly up-regulated transcripts (p-value < 0.05, FC ≥ +1.3), red: significantly down-regulated transcripts (p-value < 0.05, FC ≤ -1.3). FCs are ratios between the averages of linear expression values. If the ratio is < 1, FC is calculated as the negative reciprocal of the ratio.

			**fold change TEXUS-49**	
**gene name**	**full gene name**	**probeset ID**	**µg vs. BL-TX hyp-g**	**µg SKF vs µg**
ACSL3	acyl-CoA synthetase long-chain family member 3	NM_004457	-1.931	1.936
ADAM21	ADAM metallopeptidase domain 21	NM_003813	1.318	-1.423
AKR1CL2	aldo-keto reductase family 1, member C-like 2	NM_031436	1.774	-1.865
AMPD1	adenosine monophosphate deaminase 1 (isoform M)	NM_000036	1.545	-1.799
ANAPC7	anaphase promoting complex subunit 7	BC017316	-1.364	1.386
ANGPT1	angiopoietin 1	BC029406	-1.436	1.816
ANGPT2	angiopoietin 2	AF187858	1.394	-1.329
ANXA5	annexin A5	NM_001154	-1.369	1.397
C11orf16	chromosome 11 open reading frame 16	BC027865	1.769	-1.903
C14orf119	chromosome 14 open reading frame 119	NM_017924	1.481	-1.664
C20orf50	chromosome 20 open reading frame 50	XM_933095	1.524	-1.592
C21orf100	chromosome 21 open reading frame 100	NM_145033	1.601	1.674
C2orf33	chromosome 2 open reading frame 33	CR625753	-1.405	1.482
C2orf33	chromosome 2 open reading frame 33	NM_020194	-1.399	1.436
C3orf37	chromosome 3 open reading frame 37	BC010125	-1.369	1.478
C6orf170	chromosome 6 open reading frame 170	AK125385	-1.468	1.441
CA12	carbonic anhydrase XII	BC000278	1.713	-1.894
CALCA	calcitonin/calcitonin-related polypeptide, alpha	NM_001741	2.141	-2.283
CASR	calcium-sensing receptor (hypocalciuric hypercalcemia 1, severe neonatal hyperparathyroidism)	NM_000388	1.970	-2.107

CCDC36	coiled-coil domain containing 36	NM_178173	2.782	-3.593
CCNL1	cyclin L1	AF367476	-1.618	1.638
COBL	cordon-bleu homolog (mouse)	NM_015198	1.873	-1.913
COL14A1	collagen, type XIV, alpha 1 (undulin)	NM_021110	-1.690	1.754
DNAJB13	DnaJ (Hsp40) related, subfamily B, member 13	NM_153614	1.742	-1.825
DRD2	dopamine receptor D2	AB209832	1.441	-1.350
ELMO2	engulfment and cell motility 2	NM_133171	-1.307	1.424
EVC	Ellis van Creveld syndrome	NM_153717	2.883	-4.102
FCRLM1	Fc receptor-like and mucin-like 1	AF329495	-1.322	1.548
FGF5	fibroblast growth factor 5	BC074858	-1.337	1.381
FILIP1	filamin A interacting protein 1	NM_015687	1.432	-1.492
FLJ32065	hypothetical protein FLJ32065	BC073870	-1.439	1.423
FLJ35773	hypothetical protein FLJ35773	NM_152599	1.432	1.397
FLJ39653	hypothetical protein FLJ39653	NM_152684	1.738	-1.671
GCNT2	glucosaminyl (N-acetyl) transferase 2, I-branching enzyme (I blood group)	NM_001491	-1.399	1.325
GLS2	glutaminase 2 (liver, mitochondrial)	NM_013267	-1.524	1.578
GLT8D3	glycosyltransferase 8 domain containing 3	XM_290597	-1.500	-1.429
GOT1	glutamic-oxaloacetic transaminase 1, soluble (aspartate aminotransferase 1)	BC000498	-1.344	1.441
GSG2	germ cell associated 2 (haspin)	NM_031965	-1.428	1.411
HCP5	HLA complex P5	CR626077	1.399	-1.749
HELT	Hey-like transcriptional repressor	NM_001029887	1.405	1.455
HEMGN	hemogen	NM_018437	1.338	-1.324
HSF2	heat shock transcription factor 2	BC005329	-1.845	2.038
IGSF4B	immunoglobulin superfamily, member 4B	BC033819	1.904	-2.062
IL1RAP	interleukin 1 receptor accessory protein	NM_134470	-1.909	1.851
IPO9	importin 9	AK001264	-1.422	1.435
KCNA2	potassium voltage-gated channel, shaker-related subfamily, member 2	NM_004974	2.993	-4.167
KCNJ11	potassium inwardly-rectifying channel, subfamily J, member 11	BC064497	1.586	-1.540
KIAA0672	KIAA0672 gene product	AK023797	1.454	-1.458

LAMA4	laminin, alpha 4	BC066552	1.394	-1.318
LCE5A	late cornified envelope 5A	NM_178438	1.805	-1.949
LNX1	ligand of numb-protein X 1	BC022983	1.391	-1.669
LOC129881	hypothetical LOC129881	XM_926895	1.487	1.678
LOC136288	hypothetical protein LOC136288	XM_059832	1.406	-1.375
LOC284395	hypothetical protein LOC284395	XM_378787	1.306	-1.384
LOC392269	similar to Transcription factor SOX-2	XM_928112	2.567	-2.651
LOC441818	similar to WW domain binding protein 11	XM_929771	-1.403	1.423
LOC642578	similar to acyl-CoA synthetase medium-chain family member 1	XM_926063	1.322	1.803
LOC642867	hypothetical protein LOC642867	XM_931130	1.355	-1.521
LOC643824	similar to cauxin	XM_933277	1.344	1.376
LOC644277	hypothetical protein LOC644277	XM_932098	2.157	-2.255
LOC644280	hypothetical protein LOC644280	XM_497769	1.379	1.379
LOC644320	hypothetical protein LOC644320	XM_934362	1.338	1.350
LOC644541	hypothetical protein LOC644541	XM_932234	1.308	1.539
LOC644629	hypothetical protein LOC644629	XM_932271	1.407	-1.809
LOC645158	hypothetical protein LOC645158	XM_932692	-1.499	1.555
LOC645343	hypothetical protein LOC645343	XM_928377	1.442	1.390
LOC645491	similar to phosphoglucomutase 5	XM_928516	1.628	-1.760
LOC645903	hypothetical protein LOC645903	XM_933255	1.567	-1.620
LOC646055	hypothetical protein LOC646055	XM_933369	2.002	-1.834
LOC646522	hypothetical protein LOC646522	XM_933676	2.000	-2.370
LOC653522	similar to hypothetical protein FLJ22679	XM_927884	-1.415	1.539
LOC653555	similar to Charged multivesicular body protein 4b (Chromatin modifying protein 4b) (CHMP4b)	XM_930357	-1.357	1.389
LRRC28	leucine rich repeat containing 28	AY358082	-1.328	1.386
LYSMD4	LysM, putative peptidoglycan-binding, domain containing 4	BX640928	1.397	-2.045
MCM3AP	MCM3 minichromosome maintenance deficient 3 (S. cerevisiae) associated protein	NM_003906	-1.490	1.563
MDM1	Mdm4, transformed 3T3 cell double minute 1, p53 binding protein (mouse)	NM_020128	-1.500	1.695

MGST2	microsomal glutathione S-transferase 2	BC025416	-1.475	1.352
MS4A6E	membrane-spanning 4-domains, subfamily A, member 6E	BC069322	-1.393	1.373
MYRIP	myosin VIIA and Rab interacting protein	BC109312	-1.517	1.615
NPR3	natriuretic peptide receptor C/guanylate cyclase C (atrionatriuretic peptide receptor C)	NM_000908	1.442	-1.417
PDCD1LG2	programmed cell death 1 ligand 2	BC074766	1.409	-1.411
PDCD7	programmed cell death 7	NM_005707	-1.394	1.475
PEG10	paternally expressed 10	BC050659	1.332	-1.370
PFDN6	prefoldin subunit 6	NM_014260	-1.399	1.338
PGEA1	PKD2 interactor, golgi and endoplasmic reticulum associated 1	NM_015373	-1.470	1.516
POU2F1	POU domain, class 2, transcription factor 1	BC001664	-1.354	1.460
PPP6C	protein phosphatase 6, catalytic subunit	NM_002721	-1.477	1.472
PRH2	proline-rich protein HaeIII subfamily 2	NM_005042	-1.333	1.308
RACGAP1	Rac GTPase activating protein 1	BC032754	-1.453	1.362
RASSF5	Ras association (RalGDS/AF-6) domain family 5	NM_031437	-1.387	1.327
RNF185	ring finger protein 185	NM_152267	-1.372	1.400
RTP2	receptor transporter protein 2	NM_001004312	1.352	-1.709
SEMG1	semenogelin I	NM_198139	1.576	-1.512
SETD4	SET domain containing 4	NM_001007259	-1.411	1.423
SFRS15	splicing factor, arginine/serine-rich 15	AL834304	-1.425	1.512
SLC4A9	solute carrier family 4, sodium bicarbonate cotransporter, member 9	AF332961	1.405	1.438
SLC6A11	solute carrier family 6 (neurotransmitter transporter, GABA), member 11	NM_014229	1.796	-1.893
TCEB3	transcription elongation factor B (SIII), polypeptide 3 (110 kDa, elongin A)	NM_003198	-1.325	1.416
TDP1	tyrosyl-DNA phosphodiesterase 1	BC015474	-1.489	1.508
TGFBI	transforming growth factor, beta-induced, 68 kDa	NM_000358	-1.374	1.383
TPD52L2	tumor protein D52-like 2	NM_003288	-1.458	1.466
TPM1	tropomyosin 1 (alpha)	AY640414	-1.431	1.464
TRH	thyrotropin-releasing hormone	BC074888	1.592	-1.539
TRPM8	transient receptor potential cation channel, subfamily M, member 8	NM_024080	-1.472	1.364

VWCE	von Willebrand factor C and EGF domains	NM_152718	2.357	-2.557
WIT1	Wilms tumor upstream neighbor 1	NM_015855	1.436	1.470
WWOX	WW domain containing oxidoreductase	BC003184	1.323	-1.804
ZNF16	zinc finger protein 16 (KOX 9)	NM_001029976	-1.301	1.311
ZNF226	zinc finger protein 226	NM_001032375	-1.548	1.857
ZNF275	zinc finger protein 275	XM_931920	-1.433	1.460
ZNF275	zinc finger protein 275	XM_931925	-1.431	1.501
ZNF283	zinc finger protein 283	AK098175	-1.329	1.541

Supplementary Table S2: Regulation of transcripts involved in apoptotic/necroptotic pathways in response to altered gravitational conditions in the 19th DLR parabolic flight campaign. Hypergravity-sensitivity: Differentially expressed in BL-PFC hyp-g vs 1g IF, but not differentially expressed in 1g IF vs H/W 1g GC. Microgravity sensitivity: Differentially expressed in µg vs BL-PFC hyp-g, but not differentially expressed in 1g IF vs H/W 1g GC or in the same direction in BL-PFC hyp-g vs 1g IF. Differential expression is defined as t-test p-value < 0.05; fold change ≤ -1.3 or ≥ +1.3.

	**hypergravity sensitivity**	**microgravity sensitivity**
not differentially expressed	**444 (80%)**	**549 (99%)**
up-regulated	45 (8%)	1 (<1%)
down-regulated	64 (12%)	3 (<1%)
**total number**	**553**	**553**

Supplementary Table S3: Regulation of selected key molecules involved in apoptotic/necroptotic pathways in response to altered gravitational conditions in the 19th DLR parabolic flight campaign. All probeset-IDs that correspond to a gene are listed. Bright blue: not significantly up-regulated transcripts (p-value > 0.05, FC ≥ +1.3). Yellow: not differentially expressed (FC ≥ -1.3 and ≤ +1.3, p-value independent). Orange: not significantly down-regulated transcripts (p-value > 0.05, FC ≤ -1.3). Red: significantly down-regulated transcripts (p-value < 0.05, FC ≤ -1.3). FCs are ratios between the averages of linear expression values. If the ratio is <1, FC is calculated as the negative reciprocal of the ratio.

		**hypergravity-sensitive transcripts** comparison BL-PFC hyp-g vs 1g IF			**microgravity-sensitive transcripts** comparison µg vs BL-PFC hyp-g		
gene	probeset-ID	t-test p-value	fold change	tendency	t-test p-value	fold change	tendency
Caspase 3	NM_004346	0.182	-1.30	↓	0.624	1.14	↔
Caspase 7	NM_001227	0.189	-1.25	↔	0.427	1.20	↔
Caspase 8	AF422925	0.064	-1.52	↓	0.495	1.24	↔
	BC068050	0.042	-1.86	↓	0.364	1.44	↑
	NM_033358	0.007	-1.96	↓	0.199	1.50	↑
	NM_001228	0.096	-1.63	↓	0.610	1.23	↔
Rip1	NM_003804	0.575	-1.13	↔	0.645	1.17	↔
Rip3	AY494982	0.609	-1.11	↔	0.873	-1.05	↔
	AY494983	0.393	-1.11	↔	0.846	1.03	↔
	BC041668	0.218	1.22	↔	0.376	-1.16	↔
	BC062584	0.462	-1.15	↔	0.634	1.14	↔
	NM_006871	0.710	-1.06	↔	0.916	-1.03	↔
FADD	BC000334	0.050	-1.40	↓	0.317	1.26	↔
	NM_003824	0.118	-1.28	↔	0.485	1.16	↔

Supplementary Table S4: Regulation of transcripts involved in apoptotic/necroptotic pathways in response to altered gravitational conditions in the TEXUS-49 suborbital ballistic rocket campaign. Hypergravity sensitivity: Comparison BL-TX hyp-g vs H/W 1g GC. Microgravity sensitivity: Comparison µg vs BL-PFC hyp-g without trancripts that are differentially expressed in the same direction in BL-TX hyp-g vs H/W 1g GC. Differential expression is defined as t-test p-value < 0.05; fold change ≤ -1.3 or ≥ +1.3.

	**hypergravity sensitivity**	**microgravity sensitivity**
not differentially expressed	**390 (71%)**	**484 (88%)**
up-regulated	140 (25%)	7 (1%)
down-regulated	23 (4%)	62 (11%)
**total number**	**553**	**553**

Supplementary Table S5: Regulation of selected key molecules involved in apoptotic/necroptotic pathways in response to altered gravitational conditions in the TEXUS-49 suborbital ballistic rocket campaign. All probeset-IDs that correspond to a gene are listed. Dark blue: significantly up-regulated transcripts (p-value < 0.05, FC ≥ +1.3). Bright blue: not significantly up-regulated transcripts (p-value > 0.05, FC ≥ +1.3). Yellow: not differentially expressed transcripts (FC ≥ -1.3 and ≤ +1.3, p-value independent). Orange: not significantly down-regulated transcripts (p-value > 0.05, FC ≤ -1.3). Red: significantly down-regulated transcripts (p-value < 0.05, FC ≤ -1.3). FCs are ratios between the averages of linear expression values. If the ratio is <1, FC is calculated as the negative reciprocal of the ratio.

		**hypergravity-sensitive transcripts** comparison BL-TX hyp-g vs H/W 1g GC			**microgravity-sensitive transcripts** comparison µg vs BL-TX hyp-g		
gene	probeset-ID	t-test p-value	fold change	tendency	t-test p-value	fold change	tendency
Caspase 3	NM_004346	0.001	2.33	↑	0.042	-2.12	↓
Caspase 7	NM_001227	0.004	1.81	↑	0.064	-1.62	↓
Caspase 8	AF422925	0.056	1.59	↑	0.041	-1.91	↓
	BC068050	0.140	1.54	↑	0.054	-2.00	↓
	NM_033358	0.104	1.51	↑	0.061	-1.88	↓
	NM_001228	0.046	1.74	↑	0.035	-2.06	↓
Rip1	NM_003804	0.124	1.45	↑	0.129	-1.59	↓
Rip3	AY494982	0.862	-1.05	↔	0.389	-1.24	↔
	AY494983	0.633	-1.17	↔	0.569	-1.12	↔
	BC041668	0.084	-2.02	↓	0.053	2.25	↑
	BC062584	0.969	-1.01	↔	0.253	-1.30	↓
	NM_006871	0.557	1.12	↔	0.131	-1.27	↔
FADD	BC000334	0.013	-1.86	↓	0.224	-1.43	↓
	NM_003824	0.006	-1.87	↓	0.236	-1.38	↓

No strong effects on apoptosis/necroptosis-associated pathways in altered gravity

In order to investigate proposed effects of altered gravity on apoptosis[60,61], we screened our data sets for genes involved in apoptosis and necroptosis and identified 553 genes from KEGG pathways (Apoptosis - Homo sapiens PATHWAY: hsa04210; Necroptosis - Homo sapiens PATHWAY: hsa04217) and the review from Elmore[62]. 80% of transcripts involved in apoptotic and necroptotic pathways were unaltered in the hypergravity phase of the parabolic flight and 99% in the microgravity phase (Supplementary Table S2). Among the apoptosis/necroptosis-related hypergravity-sensitive transcripts, we identified 8% being up and 12% being down-regulated, whereas less than 1% of all apoptosis/necroptosis-related microgravity-sensitive transcripts was differentially regulated (Supplementary Table S2). In the suborbital ballistic rocket experiment, 71% of apoptosis/necroptosis-related transcripts were unaltered in the hypergravity phase and 88% in the microgravity phase (Supplementary Table S4). Among the hypergravity-sensitive transcripts, 25% were up regulated and 4% were down-regulated, while among the microgravity-sensitive transcripts, 1% was up-regulated and 11% were down-regulated (Supplementary Table S4). Since many genes listed in the KEGG pathways only play a secondary role, we directly analyzed a selection of key molecules of apoptosis and necroptosis: Caspase 3, caspase 7, caspase 8, rip1, rip3, and FADD (Supplementary Tables S3 and S4) and found that 20 s of hypergravity or microgravity altered the transcripts of these selected apoptosis/necroptosis relevant genes only slightly. In 20 s of mild hypergravity (1.8g), caspase 8 transcripts were decreased (Supplementary Table S3) indicating an anti-apoptotic effect, but increased after 75 s of stronger hypergravity (up to 13.5g) arguing for a pro-apoptotic properties (Supplementary Table S5). After 20 s of microgravity, two caspase 8 transcripts were slightly up-regulated (Supplementary Table 3), but down-regulated after 300 s of microgravity (Supplementary Table S5). 75 s of hypergravity resulted in a slight up-regulation (up to 2.33-fold) of all analyzed caspases, whereas the necroptosis specific genes remained mostly unaltered or were down-regulated (Supplementary Table S5). This up-regulation was completely reversed in the 300 s of the following microgravity phase. All investigated transcripts representing caspase 3, 7, and 8 were down-regulated in microgravity (comparison µg versus BL-TX hyp-g, Supplementary Table S5) and necroptosis genes were either unaltered or further down-regulated. Only rip3 that was down-regulated during the hypergravity phase was again up-regulated in microgravity (Supplementary Table S5).
